# How do astrocytes shape synaptic transmission? Insights from electrophysiology

**DOI:** 10.3389/fncel.2013.00159

**Published:** 2013-10-01

**Authors:** Glenn Dallérac, Oana Chever, Nathalie Rouach

**Affiliations:** Neuroglial Interactions in Cerebral Physiopathology, Center for Interdisciplinary Research in Biology, CNRS UMR 7241, INSERM U1050, Collège de FranceParis, France

**Keywords:** glia, neurons, neuroglial interactions, synapses, ionic channels, plasticity, dual recordings, electrophysiology

## Abstract

A major breakthrough in neuroscience has been the realization in the last decades that the dogmatic view of astroglial cells as being merely fostering and buffering elements of the nervous system is simplistic. A wealth of investigations now shows that astrocytes actually participate in the control of synaptic transmission in an active manner. This was first hinted by the intimate contacts glial processes make with neurons, particularly at the synaptic level, and evidenced using electrophysiological and calcium imaging techniques. Calcium imaging has provided critical evidence demonstrating that astrocytic regulation of synaptic efficacy is not a passive phenomenon. However, given that cellular activation is not only represented by calcium signaling, it is also crucial to assess concomitant mechanisms. We and others have used electrophysiological techniques to simultaneously record neuronal and astrocytic activity, thus enabling the study of multiple ionic currents and in depth investigation of neuro-glial dialogues. In the current review, we focus on the input such approach has provided in the understanding of astrocyte-neuron interactions underlying control of synaptic efficacy.

## Introduction

Dynamic bidirectional communication between astrocytes and neurons is now thought to contribute to brain information processing. Indeed, astrocytes are equipped to sense and integrate neuronal information through ionic channels, neurotransmitter receptors and transporters and intracellular signaling pathways. Such machinery is reported to endow astrocytes with the ability to modulate neurotransmission through a variety of mechanisms involving morphological plasticity and uptake or release of numerous neuroactive factors. For instance, astrocytes regulate the formation and stability of synapses, receptor trafficking and the moment-to-moment synaptic activity by releasing various molecules such as proteoglycans, cytokines, energy metabolites or neuromediators, as described in several recent comprehensive reviews (Perea et al., 2009; Giaume et al., 2010; Hamilton and Attwell, 2010; Ben Achour and Pascual, 2012; Nedergaard and Verkhratsky, 2012; Santello and Volterra, 2012). They can also modify the efficacy of synapses by controlling extracellular glutamate concentration via transporters mediating clearance of neurotransmitters (Oliet et al., 2001) or by changing the extracellular space volume (Piet et al., 2004) through a plastic physical coverage of neurons. Although the repertoire of such astroglial regulations of neurotransmission continuously expands, their detailed cellular and molecular mechanisms, as well as their occurrence at certain developmental stages during physiological or pathological conditions remain unclear. Indeed, as neurons and astrocytes use many similar transmitters, receptors and transporters, thereby limiting utilization of a gliotransmission selective pharmacology, the specific contribution of astrocytes to synaptic transmission and plasticity has been difficult to demonstrate directly. Several approaches have been used to tackle the involvement of astrocytes in neuronal activity, ranging from molecular biology, pharmacology, imaging, electrophysiology to behavioral studies. The main difficulties in the field of glia research lie in developing experimental tools allowing to selectively perturb astroglial functions, as well as choosing the best readout to assign specific neuromodulatory functions to astrocytes.

Astrocytes are considered to be electrically non-excitable cells, as they do not fire action potentials. Thus, in the last decades, most of the attention in the glial community has focused on imaging and altering dynamic calcium (Ca^2+^) signaling of astrocytes, proposed to represent their excitability and to mediate neuromodulatory actions of astrocytes. However, astrocytes are not electrically silent cells, and pioneer *in vivo* intra-glial electrophysiological recordings in the sixties revealed their peculiar cellular dynamic profile (Phillips, 1956; Sugaya et al., 1964; Karahashi and Goldring, 1966; Castellucci and Goldring, 1970; Ransom and Goldring, 1973). Astrocytes indeed exhibit unique biophysical and functional electrical properties, sensitive to neuronal activity and capable of modulating neurotransmission. Thus, electrophysiological recordings of activity-dependent astroglial and neuronal responses have unexpectedly turned out to be a powerful method to unravel online the dynamics of neuroglial ionic signaling. In the current review, we focus on how electrophysiological recordings have provided unique quantitative information about the membrane properties of astrocytes, and the active ionic neuroglial dialog involved in information processing. Limitations and future directions on the use of such technique in the field of neuroglial research are also discussed.

## Astrocytic properties determined by electrophysiological recordings

### Biophysical membrane properties of astrocytes

#### Astroglial membrane properties in vivo

Mature glia recorded *in vivo* with sharp electrodes, mainly identified as astrocytes in the gray matter (Mishima et al., 2007), display homogeneous, specific and easily identifiable properties: a hyperpolarized resting membrane potential (~–80 mV) and low input resistance (~4–20 MΩ) and capacitance (~10–25 pF) compared to neurons (Amzica and Neckelmann, 1999; Amzica, 2002; Amzica and Massimini, 2002; Amzica et al., 2002; Seigneur et al., 2006; Mishima et al., 2007; Mishima and Hirase, 2010). The astroglial membrane potential is close to the nernstian equilibrium for potassium ions (E_K_), thus reflecting the presence of high resting conductances for potassium ions (K^+^) (Somjen, 1975). Furthermore, although no action potential or synaptic event can be recorded or induced by depolarizing pulses, astrocytic membranes are animated by very slow fluctuations that are intimately related to changes in neuronal activities. Indeed, due to their strong K^+^ conductances, astrocytes are highly sensitive, with a quasi-nernstian relationship, to changes in extracellular K^+^ levels associated with neuronal activity (Amzica, 2002; Amzica and Massimini, 2002) (Figure 3B).

**Figure 1:**
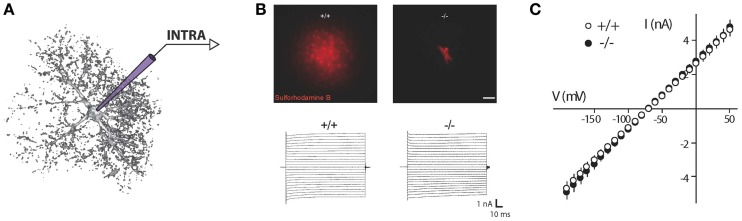
**Recording of astroglial membrane properties. (A)** Schematic representation of intracellular (whole cell patch clamp recording or sharp electrode) recording of an astrocyte. **(B)** Upper panel: dye coupling experiments show tens of coupled cells after patching of a single astrocyte with an intra-pipette solution containing sulforhodamine-B (red). Knockout mice for astroglial connexins (Cx30^−/−^Cx43^fl/fl^ hGFAP-cre) exhibit a total absence of astrocytic gap junctional coupling. Lower panel: to determine astroglial membrane resistance in a whole cell patch clamp configuration, short incremental voltage pulses are imposed to the astroglial membrane clamped at −80 mV, and evoked currents are recorded. **(C)** Quantification of the current/voltage relationship (IV curve). Illustration depicting current responses recorded in a hippocampal astrocyte from wild type and astroglial connexins knockout (Cx30^−/−^Cx43^fl/fl^ hGFAP-cre) animals. Both groups display a quasi-ohmic profile of the IV curve, with a similar slope, indicating a comparable membrane resistance. Adapted, with permission, from Pannasch et al. (2011) **(B,C)**.

Such properties are also used to identify mature astrocytes in slices (Zhou, 2005). Classically, membrane resistances of astrocytes is determined by quantifying the current/voltage (IV) relationship, i.e., the current readout in response to voltage incremental impositions or voltage response to current injections through whole cell recording pipettes. Because of the linear, quasi-ohmic profile of the IV curve usually recorded in astrocytes, these cells are conventionally considered as passive (Figure 1). This same electrophysiological profile can also be found in juvenile tissues, irrespective of brain structure and animal species (Chvátal et al., 1995; Matthias et al., 2003; Grass et al., 2004; Wallraff et al., 2004; Isokawa and McKhann, 2005; Djukic et al., 2007; Adermark and Lovinger, 2008; Kafitz et al., 2008; Même et al., 2009; Pannasch et al., 2011).

#### Major resting conductances sustaining hyperpolarized membrane potential

High K^+^ permeability of glial cell membranes is a major characteristic discovered in the late sixties by Orkand and Kuffler (Kuffler et al., 1966; Orkand et al., 1966). For decades, the nature of such conductance remained unknown. Major insights in this field have been obtained by pioneer work on retinal Müller cells (Newman and Reichenbach, 1996). Since then, numerous studies based on pharmacological approaches or using transgenic animals have now provided strong evidences that the inward rectifier K_ir_4.1 channel, the main K_ir_ channel expressed in astrocytes (Olsen et al., 2006), is responsible for the main astroglial K^+^ conductances. The latter were first observed *ex vivo* (Neusch et al., 2006; Olsen et al., 2006; Djukic et al., 2007; Kucheryavykh et al., 2007; Bay and Butt, 2012) and subsequently confirmed *in vivo* (Chever et al., 2010). Also, two-pore K^+^ channels (K2P) participate to astroglial K^+^ conductances (Zhou et al., 2009), yet secondarily to K_ir_4.1 channels (Seifert et al., 2009; Zhou et al., 2009).

High membrane permeability for K^+^ is tightly linked to the degree of hyperpolarization of mature astrocytes. Indeed during development, hyperpolarization of the astrocytic membrane, progressively approaching a resting potential close to E_K_, and the establishment of highly passive conductances occurs in parallel. Such property has been attributed to a progressive increase in K_ir_4.1 channel expression (Bordey and Sontheimer, 1997; Kalsi et al., 2004). Furthermore, specific deletion of K_ir_4.1 or pharmacological blockade of K_ir_ channels leads to a pronounced depolarization of astrocytes (Neusch et al., 2006; Olsen et al., 2006; Djukic et al., 2007; Chever et al., 2010) and to membrane potential fluctuations that fail to reflect changes in extracellular K^+^ levels (Figure 3C) (Chever et al., 2010), indicating that K_ir_4.1 are responsible for the main resting K^+^ conductance of astroglial membranes.

Given the major impact these channels have on astroglial membrane K^+^ permeability, K_ir_4.1 invalidation is expected to result in a severe reduction of passive conductances; this has been observed, but to a much lower extent than anticipated (Neusch et al., 2006; Olsen et al., 2006; Djukic et al., 2007), thus pointing out the limits of IV protocols for analysis of intrinsic membrane properties from mature astrocytes *in situ*. Indeed, conductances revealed by IV protocols are not necessarily those involved in the resting state of astrocytes. The general viewpoint is that K_ir_4.1 channels are enriched in distal processes of astrocytes, and therefore do not contribute to the major conductances measured from the cell body. Considering the reduced space clamp of astrocytic membranes (Zhou et al., 2009), patch clamp of astroglial processes would be more adapted to specifically assess K_ir_4.1 functions.

#### Implication of gap junctional conductances in membrane properties

Astrocytes are interconnected through pores called gap junctions that enable passage of various small molecules (<1.5 kDa), hence implying ionic and metabolic coupling between cells. Biochemical intercellular transfer (such as glucose or ATP) has been extensively studied using passive dyes loaded in the astrocytic network through a patch pipette (Giaume et al., 2012). Complementary information has also been obtained in culture using paired recording of connected astrocytes, allowing assessment of gap junctional conductances; in weakly coupled pairs, even single gap junctional channel events could be detected (Giaume et al., 1991). Such approach has proven difficult to perform in acute slices, as in these conditions, astrocytes present larger passive properties and exhibit substantially more intercellular connections (Même et al., 2009; Xu et al., 2010). Traditionally, the low passive resistance of mature astrocytes has been attributed to their extensive gap junction mediated coupling. However, such conclusion is mostly based on pharmacological inhibition of gap junctional communication, using agents that are well-known for their poor selectivity (e.g., acidification, anesthesics, alcohols, endothelins, carbenoxolone). Their application on slices has been shown to decrease astrocytic conductances without affecting the general passive pattern (Wallraff et al., 2004; Schools et al., 2006; Adermark and Lovinger, 2008). Furthermore, it has been recently shown that treatment of slices with gap junction blockers would facilitate the analysis of non-linear profiles (Seifert et al., 2009; Olsen, 2012), placing astrocytic gap junctional coupling as a major current leak. However, whilst gap junctional communication mainly occurs between astrocytic processes, excised outside-out patch recordings from soma show intact passive current profile, suggesting that the major conductances subtending the linear profile of IV relationships are not located in processes. This result is further supported by double patch clamp recordings performed on the same astrocytic soma, showing that 80% of the current injected through one electrode is lost when recorded *via* the second electrode (~5 μm away) (Zhou et al., 2009). Finally, selective deletion of the main connexins expressed in astrocytes, connexin 43 and 30, leading to a complete loss of intercellular dye coupling, only weakly reduces (Wallraff et al., 2006) or leaves intact (Pannasch et al., 2011) the membrane resistance of astrocytes (Figures 1B,C). Altogether, these studies indicate that astroglial gap junctional coupling plays a minor role, if any, in astroglial low membrane resistance and somatic passive IV relationship.

### Astroglial functional channels

Considering astrocytes as passive cells that mainly express leak K^+^ channels and only undergo passive membrane potential fluctuations is an old fashioned simplistic view. Astrocyte membranes are composed of a large variety of ion channels, transmitter receptors and transporters (Barres, 1991; Sontheimer, 1994; Verkhratsky and Steinhäuser, 2000). Intensive characterization of astroglial electrophysiological properties using patch clamp recordings has revealed in the past decades diverse profiles, depending on the brain region and developmental stage investigated, unraveling the complexity of astrocytic populations. Moreover, and above all, a variety of functional channels with dynamic expression patterns has been reported.

#### Dynamic and evolution of expression patterns

Astrocytes express at high density a whole range of channels, including K^+^, Na^+^, Cl^−^, or Ca^2+^ permeable channels (Steinhäuser et al., 2013). The ionic channel expression pattern of astrocytes is dominated by K^+^ channels, of which the relative contribution to the general conductances evolves with cell maturation (Sontheimer and Waxman, 1993; Kressin et al., 1995; Bordey and Sontheimer, 1997; Zhou, 2005). It is generally assumed that differentiation into mature astrocytes takes place within the first 2–3 weeks after birth (Nixdorf-Bergweiler et al., 1994; Zhou, 2005). In immature tissues, the majority of astrocytes displays prominent delayed K^+^ outwardly rectifiers (K_D_) and transient “A” type currents (K_A_), which dominate whole cell electrophysiological pattern. With time, more astrocytes progressively express K_ir_ channels and the large inward rectifier currents overwhelm all other K^+^ conductances. This change in expression profile is accompanied by a hyperpolarization of the membrane potential, a large increase in the general cellular conductance and a quasi-linear IV curve. Such change in electrophysiological pattern is characteristic of a mature, differentiated astrocyte, and has been suggested to be at the origin of cell proliferation arrest (Olsen and Sontheimer, 2008). Na^+^ channels have also been detected in astrocytes (Kressin et al., 1995; Bordey and Sontheimer, 1997), although their relative conductance is too weak, compared to K^+^ channels, to trigger action potentials (Sontheimer and Waxman, 1992; Bordey and Sontheimer, 1997). Thus, astroglial Na^+^ likely serves other functions (Sontheimer et al., 1996).

#### Implication in pathology

Characterization of channel expression developmental patterns in astrocytes has been particularly helpful to understand modifications affecting astrocytes in various pathologies. Indeed, the electrophysiological phenotypes of astrocytes are severely altered in gliomas (Ransom et al., 2001), reactive gliosis associated with brain insult (MacFarlane and Sontheimer, 1997), cortical dysplasia (Bordey et al., 2001) or epilepsy (Bordey and Sontheimer, 1998; O'Connor et al., 1998; Schröder et al., 2000), and curiously indicate reprogramming into an immature current pattern. Some common characteristics are a decrease or abolishment of K_ir_ conductances, in some cases an up-regulation of Na^+^ conductances, and overall a drastic dominance of K^+^ outward conductances, directly linked to cellular proliferation (Olsen and Sontheimer, 2008; Molenaar, 2011). Numerous questions, however, remain open, notably how these changes in channel expression affect cell functions.

Assessment of electrophysiological cellular profiles in slices or in cultures allows unraveling functional properties of channels expressed at astrocytic membranes, appreciating their weight with regard to the general cell conductance, and exploring their specific properties in terms of kinetics and modulations. However, deciphering the physiological implications of channels that do not prevail in mature astrocytes is more complicated, especially in slices, because of the predominance of K_ir_ conductances and technical limitations such as low space clamp.

Numerous channels, such as K_D_ or K_A_ are probably not open at resting membrane potential, because their activation voltage thresholds are far from resting membrane potential (Bordey and Sontheimer, 1997), and the latter only changes by a few millivolts upon neuronal activation in a physiological context. Thus, these voltage gated ion channels are likely to be primarily active in pathological conditions where astrocytes reach aberrant depolarized states during paroxysmal events such as seizures or following trauma, hypoxia or ischemia (Somjen, 1975). This presumption is however extrapolated from whole cell recordings, where astroglial membrane isopotentiality is assumed. Recordings from soma are most certainly not adequate to investigate fine electrical compartments where particular sets of channels might be functional in specific microdomains.

#### Are they all astrocytes?

Caution is needed when considering past developmental studies characterizing astroglial channel pattern, because some of them were performed at a time where the complexity of the glial lineage was not yet clearly set. An attempt to develop a classification of astrocytes according to their pattern of protein expression and electrophysiological profiles has enabled, in the last years, to define a new class of glial cells. First called “complex cells” by opposition to “passive cells,” they were initially identified as astrocytes in patch clamp investigations on slices from immature or juvenile animals (Steinhäuser et al., 1994; Kressin et al., 1995; Bordey and Sontheimer, 1997). Later renamed NG2 cells, in reference to the NG2 proteoglycan expressed on their surface, these peculiar cells exhibit numerous distinct morphological and functional properties compared to astrocytes. This includes in particular their lack of interconnection through gap junction channels and their functional expression of voltage dependent K^+^, Na^+^, and Ca^2+^ conductances, as well as ionotopic GABA and glutamate receptors (Lin and Bergles, 2002; Matthias et al., 2003; Wallraff et al., 2004). Interestingly, this new class of cells is also detectable in the adult brain (Zhou, 2005). NG2 cells acquire with maturation a linear electrophysiological profile, which closely parallels myelinization (Maldonado et al., 2013). They are oligodendrocyte precursor cells, i.e. a class of progenitors that generate myelinating oligodendrocytes during development and after demyelinating injury in the mature brain (Richardson et al., 2011). A new field of investigation is now emerging to decipher specific properties and functions of NG2 cells in developing and mature brain. The specific interactions of this novel neuroglia with astrocytes and neurons should be, in the forthcoming years, of particular interest (Bergles et al., 2010).

## Monitoring astrocyte functions with electrophysiology

**Figure 2:**
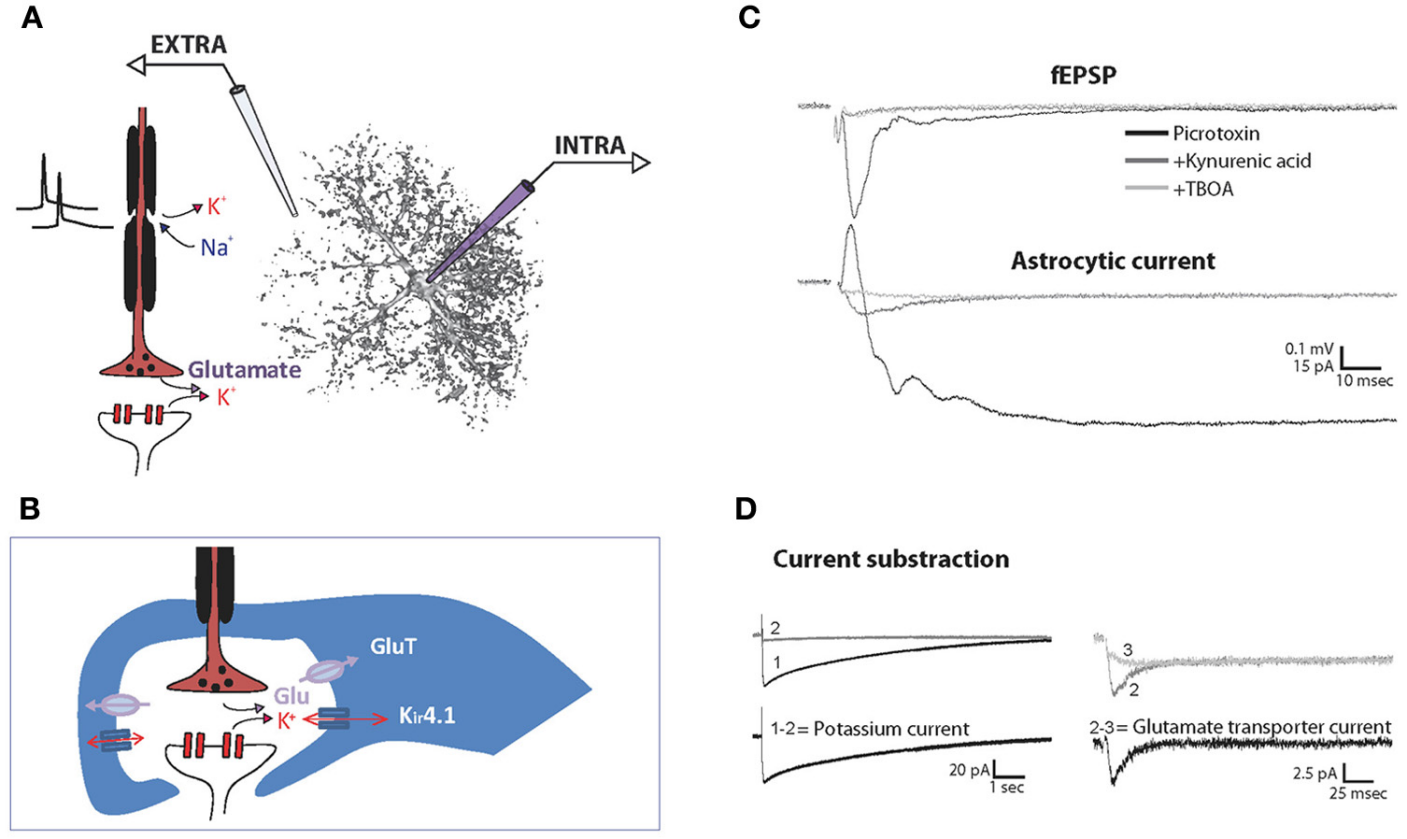
**Recording of synaptically evoked GLT and K^+^ currents in astrocytes.** Schematic representation of the experimental setting for simultaneous recording of extracellular fEPSP (EXTRA) and intracellular astrocytic currents (INTRA) **(A)** and of the main channels and transporters involved in the synaptically evoked astroglial currents at the tripartite synapse **(B)**. Simultaneous recordings of hippocampal fEPSP and astroglial currents evoked synaptically by single stimulation in basal conditions, after application of the ionotropic glutamate receptor blocker kynurenic acid, and further application of the GLT antagonist TBOA. To isolate excitatory currents, the GABA_A_ receptor blocker picrotoxin is present throughout the experiment **(C)**. Note that the initial fast outward current component in the presence of picrotoxin reflects fEPSP generated by adjacent pyramidal cells. Astroglial K^+^ currents are isolated by subtracting currents remaining after kynurenic acid application (2) to total evoked currents (1). Astroglial GLT currents are then isolated by further subtraction of the currents remaining after TBOA application (3) **(D)**. Adapted, with permission from Pannasch et al. (2012b) **(C,D)**.

Astrocytes are part of the tripartite synapse and endorse important functions, among which supporting adequate neurotransmission. They undertake several functions at the level of excitatory synapses: they buffer K^+^ released by neurons, efficiently uptake glutamate and release gliotransmitters. Neuromodulation by astrocytes has been investigated using different approaches, such as recordings of neurons whilst activating or inhibiting specific astroglial pathways. Interestingly, glutamate transporter (GLT, comprising GLT-1 and/or GLAST subtypes) uptake and K^+^ buffering can be monitored directly by intra-astroglial recordings as these are the major currents that can be elicited by neuronal stimulations (Figure 2). They have been described in astrocytes from many structures, including the hippocampus (Bergles and Jahr, 1997; Araque et al., 2002; Ge and Duan, 2007; Meeks and Mennerick, 2007), the spinal cord (Zhang et al., 2009), the olfactory bulb (De Saint Jan, 2005), the cortex (Bernardinelli and Chatton, 2008; Unichenko et al., 2012), the optic nerve (Kuffler et al., 1966), the striatum (Goubard et al., 2011), the thalamus (Parri et al., 2010) and in more specialized glia such as Bergmann cells of the cerebellum (Clark and Barbour, 1997; Bellamy and Ogden, 2005). The profile and magnitude of these currents vary among studies, mainly because of protocol heterogeneity and glial diversity between brain regions. In the following section, we highlight the input electrophysiological approaches have provided in our understanding of astrocytic functions.

### Potassium flow through astrocytic membranes

#### Major contribution of K_ir_4.1 channels

Astrocyte networks ensure K^+^ homeostasis, uptaking excess extracellular K^+^ following neuronal activity, and thereby controlling neuronal excitability. Several co-transporters and K^+^ permeable channels are involved in this function, mainly Na^+^/K^+^ ATPases pumps, K_ir_ channels and Na^+^/K^+^/2Cl^−^ transporters (Amedee et al., 1997; Somjen, 2002; Kofuji and Newman, 2004; Butt and Kalsi, 2006). The implication of K2P channels has also been reported (Päsler et al., 2007; Zhou et al., 2009). Astroglial K_ir_ channels, and especially K_ir_4.1 channels, are believed to extensively participate to K^+^ buffering, allowing excess of extracellular K^+^ to flow into astrocytes. This has been suggested by several lines of evidence: (1) developmental up-regulation of K_ir_4.1 channels is correlated with maturation of K^+^ regulation mechanisms (Connors et al., 1982; Gabriel et al., 1998); (2) K_ir_4.1 are the main K_ir_ channels expressed in astrocytes (Olsen et al., 2006) and are highly expressed in astroglial perisynaptic processes (Higashi et al., 2001; Hibino, 2004), presumably in regard to hotspots of K^+^ release; (3) K_ir_4.1 channels underlie the major component of K^+^ astrocytic currents (De Saint Jan, 2005; Djukic et al., 2007); (4) the regulation of extracellular K^+^ excess is less efficient in tissues from K_ir_4.1 knockout than wild type mice (Neusch et al., 2006; Chever et al., 2010; Haj-Yasein et al., 2011; Bay and Butt, 2012).

#### Synapse as a major source of K^+^ for astrocytes

Astrocytic currents evoked in slices are mainly mediated by K^+^ conductances. Astrocytes display large and long lasting currents elicited in response to neuronal activities, which largely exceed the timescale of neuronal events. As illustrated in Figure 2C, whilst the postsynaptic response to single stimulation occurs within 20–40 ms following presynaptic activation, the astrocytic response is more than 200 times longer. In voltage clamp mode, this inward current is almost totally abolished by extracellular application of barium or by knocking out the K_ir_4.1 gene (Djukic et al., 2007), thus indicating a major contribution of K^+^ conductances. Astrocytic currents are induced by neuronal activity, as they are totally abolished by tetrodotoxin (Bergles and Jahr, 1997), and more precisely, depend on synaptic activation since they are abrogated by Cd^2+^ or total depletion of extracellular Ca^2+^, both of which prevent presynaptic release of vesicles (Bergles and Jahr, 1997; De Saint Jan, 2005). Furthermore, postsynaptic blockers of excitatory transmission are commonly used to block K^+^ currents evoked in astrocytes (De Saint Jan, 2005; Ge and Duan, 2007; Pannasch et al., 2011). Altogether, these studies indicate that astrocytes, through K_ir_4.1 channels densely expressed on processes at the vicinity of synapses, sense extracellular K^+^ mostly released from postsynaptic receptors. This is probably the case in areas enriched in synaptic contacts such as the *stratum radiatum*, in which most of the studies have been performed and where excitatory synapses have been determined to be the major source of K^+^ release (Pumain and Heinemann, 1985). However, non-synaptic release of K^+^ also occurs during firing of action potentials (D'Ambrosio et al., 2002; Bay and Butt, 2012). As a result, the contribution of axons and synapses to the general increase of K^+^ (Aitken and Somjen, 1986) and the associated astrocytic buffering varies between brain areas.

#### Are we recording K^+^ buffering?

**Figure 3:**
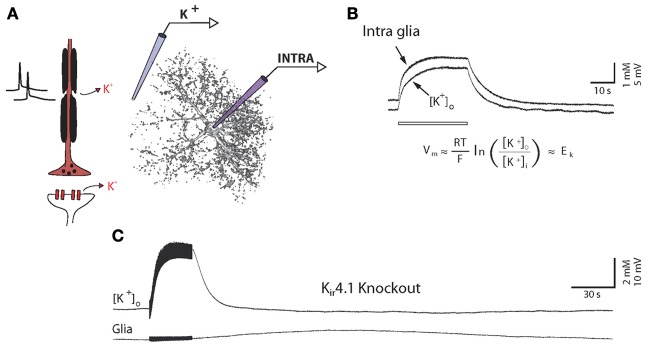
**High potassium permeability of glial cells is mediated by K_ir_4.1 channels. (A)** Schematic illustration of simultaneous recordings of extracellular K^+^ concentration (K^+^) and glial membrane potential (INTRA). **(B)** *In vivo* simultaneous recordings of extracellular K^+^ concentrations and glial membrane potential fluctuations during long lasting stimulations (10 Hz, 30 s). Glial membranes behave as K^+^ electrodes, reflecting their high K^+^ permeability. Abbreviation for Nernst equation: R, universal gas constant; T, temperature; F, Faraday constant; E_K_, K^+^ equilibrium potential; Vm, membrane potential; [K^+^]_o_, extracellular K^+^ concentration; [K^+^]_i_, intracellular K^+^ concentration. **(C)** Similar protocol applied in a K_ir_4.1 knockout animal. Glial membrane potential does not follow K^+^ fluctuations, indicating a strong loss of K^+^ conductances. A small and delayed depolarization, which is not associated with K^+^ increase, could, however, be observed in response to long lasting stimulations only. Adapted, with permission, from Chever et al. (2010) **(B,C)**.

One of the important role of astrocytes is to uptake K^+^ released by active neurons. However, considering that the K^+^ current recorded in astrocytes in voltage-clamp mode strictly corresponds to the buffered K^+^ through K_ir_4.1 channels would be a clear overestimation: as astrocytes depolarize in response to neuronal activity, a proportion of the evoked inward current usually attributed to K^+^ in astrocytes actually corresponds to the holding current needed to maintain the membrane potential at imposed voltage. Similarly, astroglial membrane potential depolarizations recorded in current clamp mode with *I* = 0 do not fully reflect K^+^ buffering, because they fluctuate according to the K^+^ equilibrium potential (E_k_) (Figure 3B), which is dynamic during neuronal activity and depends on both, intracellular and extracellular K^+^ concentrations. Consequently, to adequately assess K^+^ buffering, estimations of astroglial K^+^ intracellular concentration increase should be performed, and require simultaneous recordings of extracellular K^+^ levels and astroglial membrane potential fluctuations (Figure 3A) (Amzica et al., 2002). Alternatively, K^+^ fluorescent indicators can be used to monitor intracellular K^+^ increase in tissues (Dufour et al., 2011). Although such imaging approach can provide important spatial information about the cellular dynamics of K^+^, electrophysiology offers a considerably better temporal resolution.

#### Physiological relevance of membrane fluctuations

The high K^+^ permeability of astroglial membranes may represent a way to indirectly sense instantly changes in neuronal activity states, without energy (ATP) consumption. Whether these depolarizations are physiologically relevant *per se* has yet to be demonstrated and does represent a challenge. K^+^-mediated depolarizations have been proposed to modulate gap junction coupling (Enkvist and McCarthy, 1994; De Pina-Benabou et al., 2001; Roux et al., 2011), extracellular pH homeostasis (Chesler and Kraig, 1987, 1989) and recently rate of glycolysis (Ruminot et al., 2011). Indeed, astrocytes express a variety of channels and transporters that are sensitive to voltage changes (e.g., GLTs, Na^+^/K^+^ ATPases, channels, Na^+^/HCO3^−^ transporters), suggesting that astroglial depolarizations could favor adaptative responses to neuronal activity through changes in the functionality of these transporters and channels. Supporting this notion, small fluctuations in glial membrane potentials can indeed be recorded between UP and DOWN states of cortical slow wave sleep (Amzica and Massimini, 2002; Mishima et al., 2007). Astrocyte membrane potential also varies according to physiological (REM sleep/wakefulness vs. slow wave sleep) or pathological (coma, spreading depression, epilepsy) neuronal activity states (Amzica et al., 2002; Seigneur et al., 2006; Kroeger and Amzica, 2007). It is therefore tempting to suggest that astroglial functions and efficiency may be finely adapted to arousal and behavioral states.

### Glutamate transporters currents

Blocking the K^+^ component of evoked astroglial currents by application of ionotropic receptor antagonists unmasks a small transient current (Figure 2D). In current clamp mode, a depolarization is recorded and corresponds to an inward current in voltage clamp mode (Bergles and Jahr, 1997; Bernardinelli and Chatton, 2008). Such response has been attributed to activation of astroglial GLTs, since it is blocked by specific inhibitors such as DL-threo-β-benzyloxyaspartate (TBOA) and dihydrokainate (DHK) (Anderson and Swanson, 2000).

Among the five high affinity Excitatory Amino Acid Transporters (EAAT1-5) identified in the mammalian CNS, the two most abundant, EAAT1 (human homologue of L-glutamate/L-aspartate transporter GLAST) and EAAT2 (human homologue of glutamate transporter 1 GLT-1), are highly expressed in astrocytes. They are enriched on astrocytic processes (Rothstein et al., 1994; Chaudhry et al., 1995; Minelli et al., 2001), and are activated by an increase in extracellular glutamate concentration mainly originating from synaptic vesicular release (Mennerick and Zorumski, 1994; Bergles and Jahr, 1997). Astrocytic transporters are responsible for most of the glutamate uptake (Danbolt, 2001). Astroglial GLT currents have relatively low peak amplitude (~5–15 pA) during basal synaptic activity. Indeed, although GLTs are highly expressed on astroglial membranes (~10,000/μm^2^), show a high affinity for glutamate (EC_50_: 4–30 μM), and can bind glutamate in less than a millisecond, acting as glutamate buffers, their cycling time is low (~70 ms), and their access to synaptic glutamate is reduced, because of the marginal enwrapping of synapses by astrocytes in the CA1 hippocampal region (Tzingounis and Wadiche, 2007). Hippocampal astroglial GLTs are nevertheless responsible for most of extracellular glutamate clearance, which is otherwise performed by passive diffusion. They prevent spillover and activation of extrasynaptic receptors and neighboring synapses, as shown in numerous studies (Tzingounis and Wadiche, 2007).

The transport of one glutamate molecule is coupled to co-transport of 3 Na^+^ and 1 H^+^ ions and counter-transport of one K^+^ ion (Amato et al., 1994; Levy et al., 1998; Owe et al., 2006). According to this stoichiometry, glutamate transport is electrogenic as for each transport cycle, two net positive charges are translocated into the cell. One full cycle is composed of sequential steps that imply two conformation states: while binding of extracellular Na^+^/H^+^ ions and glutamate lead first to the translocation of glutamate from outside to inside, intracellular binding of K^+^ is associated with the reorientation of the glutamate binding site toward the outside (Tzingounis and Wadiche, 2007).

Yet, description of GLT functionality and of factors limiting transport of glutamate has enabled to unravel their key role in brain homeostasis. GLT currents have been found to be highly voltage dependent; their magnitude decreases with depolarization, becomes negligible at extreme values, but do not reverse (Barbour et al., 1991; Kirischuk et al., 2007). In addition, rate of glutamate uptake is also highly dependent on ionic environment, being primarily affected by Na^+^ (Levy et al., 1998; Kirischuk et al., 2007; Unichenko et al., 2012), K^+^ and glutamate concentrations (Brew and Attwell, 1987; Barbour et al., 1988). These limiting factors become crucially relevant with regard to pathological states. Indeed, in several pathological conditions such as ischemia and anoxia, extreme increase in extracellular K^+^ concentrations concomitant with neuronal and glial depolarizations are reported (Müller and Somjen, 2000). Because both increases in extracellular K^+^ concentration and glial depolarization reduce electrogenic glutamate uptake (Barbour et al., 1988), impaired glutamate uptake is suggested to participate to the excitotoxic rise of ambient glutamate concentrations (Jabaudon et al., 2000).

## Deciphering neuroglial interactions using astrocyte-neuron dual electrophysiological recordings

The aforementioned astrocytic properties and activities may be intrinsic features of these cells or, on the contrary, attributable to surrounding neuronal activities. Accurate assessment of astrocytic functions therefore implies simultaneous monitoring of astrocytic and neuronal activity. This can be achieved *in vitro* or *ex vivo* with fluorescent techniques allowing detection of cellular Ca^2+^ signals or with the powerful approach of using whole cell patch clamp of astrocytes coupled to neuronal recordings, allowing examination of multiple currents of different origins, as we recently described (Pannasch et al., 2012b). Such investigations have enabled to gain insights into the role and physiology of neuroglial interactions by interfering positively or negatively with astrocytic and/or neuronal function, as well as investigating the neuron-astrocyte interplay that occurs during particular activities, such as short- and long-term plasticity or in pathological conditions such as epilepsy. We shall discuss in this section how dual monitoring of neurons and astrocytes can decipher mechanisms of astroglial control of neurotransmission.

### Astroglial responses are shaped by neuronal activity

Entry of ions released during neuronal activation into astrocytes induces measurable currents of different origins. The main protagonists involved are K^+^ channels (Orkand et al., 1966; Karwoski et al., 1989; Meeks and Mennerick, 2007), glutamate and GABA transporters (Bergles and Jahr, 1997; Diamond et al., 1998; Lüscher et al., 1998; Goubard et al., 2011), as well as AMPA receptor currents in Bergmann glial cells (Clark and Barbour, 1997; Bellamy and Ogden, 2006). As discussed above, K^+^ currents are crucial to maintain the astrocyte at a markedly negative resting membrane potential, as well as for K^+^ clearance during and after neuronal activity. Glutamate and GABA transporters are essential for maintaining low concentration of extracellular neurotransmitters, thereby avoiding spillover and activation of extrasynaptic receptors and neighboring synapses (Oliet et al., 2001; Huang et al., 2004). In addition, they participate to the recycling of neurotransmitters, the glutamate-glutamine cycle and neurometabolic coupling (Anderson and Swanson, 2000; Danbolt, 2001; Huang and Bergles, 2004; Tzingounis and Wadiche, 2007).

#### Functional plasticity of neuroglial interactions

Most interestingly, triggering and recording neuronal plasticity while monitoring glial activity through a patch clamp electrode has enabled to observe short- and long-term changes of astrocytic currents that are associated to, but do not always match modifications in neuronal activity. In the cerebellum, intracellular monitoring of Bergmann cell currents, referred to as extrasynaptic currents (ESCs), revealed that glial short-term plasticity assessed using paired pulse stimulation protocols is 4 fold larger in magnitude than the well-known associated neuronal facilitation. Interestingly, such facilitation was found to be mainly mediated by AMPA receptors and GLT activation (Bellamy and Ogden, 2005). These investigators later showed using the same preparation that plasticity of cerebellar glial cells is not a simple reflection of surrounding neuronal activity. Indeed, long-term depression of GLT and AMPA receptor currents could be induced in Bergmann cells using 0.2 Hz stimulation that do not generate any change in evoked excitatory postsynaptic current (EPSC) amplitude from Purkinje neurons (Bellamy and Ogden, 2006). Using paired neuronal-glial recordings in cerebellar cultures, long-term potentiation (LTP) of Ca^2+^ permeable AMPA receptor glial currents has also been demonstrated to be inducible by prolonged 4 Hz stimulation of the granule cell. In this case, however, plasticity appeared to purely reflect changes in probability of glutamate release from the presynaptic terminal (Linden, 1997).

In the hippocampus, we and others found that single stimulation of Schaffer collaterals evoking typical fEPSP recorded in the *stratum radiatum* elicits a complex astroglial response, composed of at least two components, i.e. a long lasting K^+^ current and a transient GLT current (Bergles and Jahr, 1997; Diamond et al., 1998; Lüscher et al., 1998; Meeks and Mennerick, 2007; Bernardinelli and Chatton, 2008; Pannasch et al., 2011, 2012b). The complex astroglial current shows paired pulse facilitation (Pannasch et al., 2012b) and, noticeably, the GLT component presents a neuronal like short-term plasticity, as it reflects synaptic neurotransmitter release (Bergles and Jahr, 1997; Diamond et al., 1998; Lüscher et al., 1998; Bernardinelli and Chatton, 2008). Interestingly, such property was actually exploited to ascertain, using astrocytic patch clamp coupled to extracellular field recording, whether hippocampal long-term potentiation (LTP) is associated with a sustained increase in the probability of glutamate release. It was found that although astrocytic glutamate currents do display post-tetanic potentiation, they return to baseline within minutes, implying a postsynaptic expression of the CA1 hippocampal LTP (Diamond et al., 1998; Lüscher et al., 1998). Notwithstanding, Ge and colleagues were able to show, by maintaining stable astrocytic recordings for more than an hour and simultaneously monitoring field excitatory postsynaptic potentials (fEPSPs), a LTP like persistent facilitation of evoked currents in hippocampal astrocytes, which is abolished by an inhibitor of K^+^ channels and thus presumably mediated by K^+^ conductances (Ge and Duan, 2007). Based on our observation that most of the astroglial response to Schaffer collateral stimulation is mediated by K^+^ conductances (Pannasch et al., 2011, 2012b), we may speculate that these channels underlie the effect reported in the latter study.

**Figure 4:**
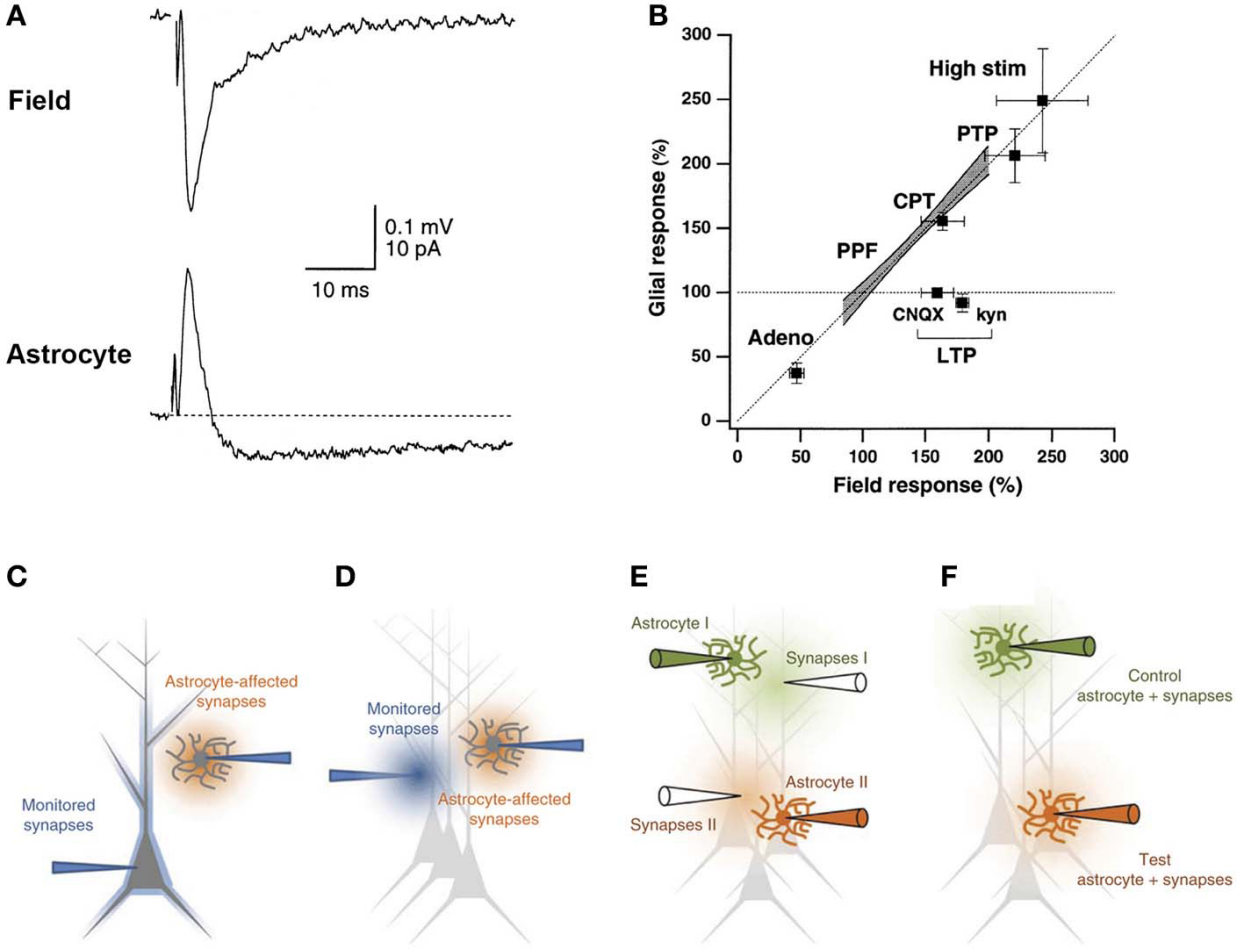
**Dual electrophysiological recording of neuronal and astroglial activities. (A)** Example showing that the extracellular field response corresponds to the initial outward signal recorded through an astrocytic patch pipette in the voltage clamp configuration. **(B)** Correlation between field and glial responses to various manipulations shows a linear relationship that falls in the line of identity. Adeno, adenosine application; PPF, paired pulse facilitation; CPT, A1 receptor antagonist CPT, High stim, high stimulation intensity. **(C–F)** Possible experimental arrangements for simultaneously monitoring neuronal and astroglial activities. **(C)** Double whole cell patch clamp experiments give access to intracellular currents for both astrocytes and neurons. **(D)** Typical electrode placement for monitoring fEPSP in parallel to astrocytic currents. **(E)** Such electrode placement can be extended to include an internal control pathway in the experimental set-up. **(F)** Monitoring the fEPSP through the astrocytic glass pipette greatly simplifies such arrangements. Adapted, with permission, from Diamond et al. (1998) **(A)**, Lüscher et al. (1998) **(B)**, and Henneberger and Rusakov (2012) **(C–F)**.

Altogether, the few investigations focusing on the plasticity of neuronal induced glial currents highlight different mechanisms depending on the plasticity considered (i.e., short- vs. long-term; potentiation vs. depression) and on the brain structure studied (e.g., cerebellum vs. hippocampus). Yet, more research taking advantage of dual electrophysiological recordings of neuronal and astroglial activities is clearly needed to fully decipher the mechanisms at play in this peculiar form of plasticity. Noteworthy, this approach being often challenging as it requires the use of multiple electrodes in a confined space, such investigations may be facilitated by the use of simultaneous monitoring of fEPSPs and astrocytic conductances through a single patch pipette as described in voltage clamp mode (Bergles and Jahr, 1997; Diamond et al., 1998; Lüscher et al., 1998) (Figures 4A,B) and recently re-validated in the current clamp configuration (Henneberger and Rusakov, 2012) (Figures 4C–F).

#### Morphological plasticity of neuroglial interactions

Astrocytes are also prone to a different form of plasticity: morphological changes in response to neuronal activity. Morphological plasticity of neuronal coverage has been demonstrated in various brain regions, including: (1) the somatosensory cortex, where stimulation of mouse whiskers resulted in significant increase in the astrocytic envelopment of excitatory synapses on dendritic spines (Genoud et al., 2006), (2) the supraoptic nuclei, which undergo pronounced reduction of the astrocytic coverage of oxytocin neurons during particular conditions such as lactation or chronic dehydration (Oliet and Bonfardin, 2010), (3) the hippocampus after LTP (Wenzel et al., 1991; Lushnikova et al., 2009), and (4) the visual cortex of rats raised in enriched environment (Jones and Greenough, 1996). Other investigations have also reported a glial swelling following electrical stimulation (MacVicar and Hochman, 1991; Hawrylak et al., 1993; MacVicar et al., 2002), most probably attributable to K^+^ buffering (Ballanyi et al., 1987). Such swelling is however meant to be transitory, as it modifies extracellular space volume and astrocytic properties (Ransom et al., 1985). Yet, in pathological condition such as epilepsy, where neuronal activity is abnormally strong, astrocytes become reactive and are swollen, so that impaired K^+^ buffering and glutamate uptake support neuronal hyperexcitability and seizure activity (Proper et al., 2002; Schröder et al., 2002). Further, using simultaneous patch clamp recording and Ca^2+^ imaging techniques, it was recently shown that initiation and maintenance of focal seizure like discharges correlate with astroglial Ca^2+^ activation, suggesting that neuronal hyperactivity engages astrocytes in a recurrent excitatory loop that promotes seizure ignition and sustains ictal events (Gómez-Gonzalo et al., 2010).

In all, analysis of morphological and functional astrocytic responses to neuronal activity have revealed that instead of being passive, these cells are, akin to neurons, able to display several forms of plasticity, of which the role remains to be identified. As exampled herein for the case of epilepsy, determining the precise function of such astrocytic adaptation to neuronal activity shall most certainly allow identifying new therapeutic targets in the numerous brain pathologies involving astrocytic inflammation and dysfunction.

### Neuronal activity is tuned by astrocytes

#### Calcium signaling

Ca^2+^ elevation represents a hallmark of cellular activation and has been shown to occur in astrocytes in response to neuronal activity (Verkhratsky and Kettenmann, 1996; Newman, 2003; Deitmer and Rose, 2010). Electrophysiology has been extensively used to impair Ca^2+^ signaling locally in populations of astrocytes through selective intracellular delivery of Ca^2+^ chelators contained in a patch pipette during whole cell recording of a single astrocyte. Such manipulation relies on the extensive connectivity of astrocytes by gap junction channels, which are permeable to Ca^2+^ chelators such as BAPTA and EGTA, enabling their diffusion in astroglial networks. This approach confirmed the importance of astrocytic Ca^2+^ rises, which trigger numerous downstream events, in the regulation of neuronal activity.

**Figure 5:**
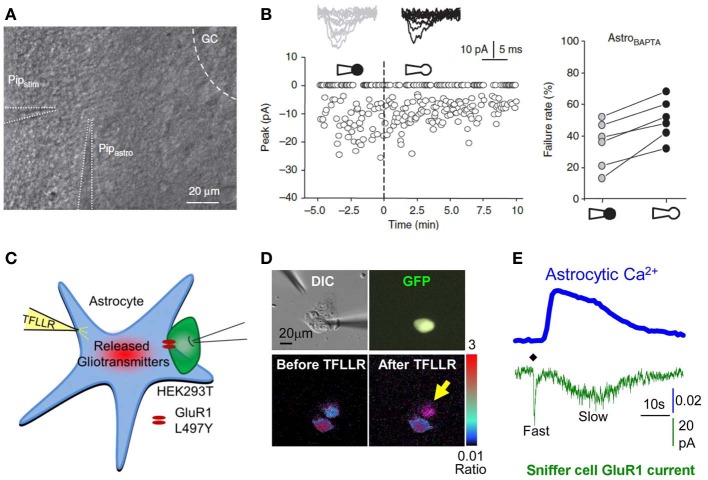
**Interfering with astrocytes influences neuronal activity. (A,B)** Infusion of BAPTA in the astrocytic network decreases synaptic efficacy in minimal stimulation conditions. **(A)** Experimental arrangement showing whole cell patch clamp of an astrocyte (Pip_Astro_) present in the dendritic tree of the recorded granule cell and 20–30 μm away from the stimulating pipette (Pip_stim_). The intra-pipette solution for patch clamp contains the Ca^2+^ chelator BAPTA. **(B)** Failure rate changes in granule cells after breaking the cell membrane and dialyzing the astrocyte with the intra-pipette solution containing BAPTA. **(C–E)** Measure of astrocytic glutamate release using the sniffer patch technique. **(C)** Schematic illustration of the sniffer-patch. The left pipette (yellow) is used for pressure application of the PAR-1 agonist TFLLR. This stimulation results in glutamate release (red cloud) producing a measureable inward current in the HEK293T sensor cell (green) expressing GluR1-L497Y containing AMPA receptors (red). **(D)** Images for sniffer patch. DIC image (upper left): two cells with two glass pipettes. GFP image (upper right): sensor cell expressing GluR1-L497Y and GFP. Pseudocolor images: Fura-2 loaded astrocyte (source cell) and sensor cells before (lower left) and after stimulation (lower right). Yellow arrow: increased Ca^2+^ in astrocyte. **(E)** Representative traces recorded from the sniffer-patch technique. Blue trace: Ca^2+^ transient recorded from astrocyte. Green trace: whole cell current recorded from sensor cell (voltage clamped at −70 mV) upon TFLLR pressure application. Diamond: TFLLR application (10 psi, 100 ms, 500 μ M). Adapted with permission from Di Castro et al. (2011) **(A,B)** and Woo et al. (2012) **(C–E)**.

In the hippocampus, astrocytic Ca^2+^ elevation has indeed been shown to up-regulate synaptic activity through several mechanisms. Di Castro and collaborators recently evidenced in the *dentate gyrus* that BAPTA infusion in the astroglial network *via* a patch pipette (Figure 5A) results in a diminished efficacy of granule cells synapses as measured intracellularly using whole cell patch clamp recording in minimal stimulation conditions (Figure 5B) (Di Castro et al., 2011). The authors attribute the Ca^2+^ rise blocked by BAPTA to activation of purinergic receptors P2Y1R, as antagonizing these leads to the same decrease in synaptic efficacy, and the presence of BAPTA in the astrocytic network occludes such effect. P2Y1Rs have recently been shown in this same hippocampal region to result in glutamate gliotransmission, activating presynaptic NMDA receptors and facilitating neurotransmitter release (Santello et al., 2011). In the CA1 area of the hippocampus, preventing astrocytic Ca^2+^ signaling using BAPTA, also reduces basal synaptic transmission efficacy, as recorded in whole cell patched pyramidal neurons subject to minimal stimulations (Panatier et al., 2011).

In contrast to the mechanism shown at perforant path-dentate gyrus synapses, Ca^2+^ rises in CA1 astrocytes during basal synaptic activity appear to mainly rely on the activation of metabotropic glutamate receptor (mGluR) mGluR5, given that application of the selective antagonist 6-methyl-2-(phenylethynyl)-pyridine (MPEP) recapitulates the increase in synaptic failures induced by BAPTA infusion in the astrocytic network, and that such chelation of astrocytic Ca^2+^ precisely occludes the MPEP effect (Panatier et al., 2011). Whether such regulation occurs in the adult brain remains to be determined, as these data were obtained from young rats (15–21 days old) and a recent investigation reports, albeit in mice, that mGluR5 signaling is barely detectable in the brain passed 3 weeks of age (Sun et al., 2013). Panatier and colleagues further demonstrate that the downstream events following astrocytic Ca^2+^ elevation include activation of presynaptic A_2A_ adenosine receptors, suggesting that in this case, gliotransmission of purines up-regulates basal synaptic activity. These important findings provide great details on the activation of hippocampal astrocytes through glutamatergic signaling.

Previous studies have also shown that astrocytes can release glutamate in response to activation of several pathways, including activation of mGluRs, but also endocannabinoid CB1 receptors or protease activated receptor PAR-1, as well as mechanical stimulation or intracellular application of inositol 1,4,5-trisphosphate (IP3), which up-regulate the frequency of slow transient NMDA currents in pyramidal neurons (Angulo et al., 2004; Fellin et al., 2004; Kang et al., 2005; Navarrete and Araque, 2008; Shigetomi et al., 2008). In line with this, work on hippocampal neurons/astrocytes cultures also reported that Ca^2+^ uncaging in astrocytes or mechanical, as well as electrical stimulation trigger glutamate gliotransmission that evokes slow inward excitatory currents in adjacent neurons (Araque et al., 1998a,b; Parpura and Haydon, 2000; Fellin et al., 2004; Navarrete et al., 2012).

CA1 fast neuronal transmission has also been found to be influenced by astroglial glutamate release subsequent to astrocytic Ca^2+^ elevation (Fiacco and McCarthy, 2004; Navarrete and Araque, 2010). In addition, the study by Henneberger and colleagues interestingly shows that clamping astrocytic free Ca^2+^ concentration at 50–80 nM, by adding 0.45 mM EGTA and 0.14 mM Ca^2+^ to the intra-pipette solution for patch clamp, impairs isolated NMDA receptor fEPSP and that such effect is due to inhibition of the NMDA receptor co-agonist D-serine gliotransmission. Remarkably, the dependence of NMDA receptor efficacy on astroglial release of D-serine was further shown to be required for synaptic plasticity, as assessed by induction of LTP (Henneberger et al., 2010). Clamping intracellular free Ca^2+^ at baseline levels is theoretically a more accurate way to assess the relevance of astrocytic Ca^2+^ activation by neuronal activities, as classical Ca^2+^ buffering most probably impairs signaling and metabolic pathways that depend on free Ca^2+^ availability. This implies that the impact of intracellular Ca^2+^ chelators on neurotransmission should be considered with great caution, and often requires confirmation of the results by using blockers of downstream pathways, such as the light chain of tetanus toxin, which blocks vesicular transmitter release.

Interestingly, to our knowledge although baseline astrocytic Ca^2+^ concentrations have been shown to lie around 50–100 nM using ratiometric Ca^2+^ indicators (Grimaldi et al., 2001; Floyd et al., 2005), no investigation as yet quantitatively reported the extent of Ca^2+^ rise following evoked synaptic transmission. Such issue is important to tackle as an intriguing recent investigation demonstrates that chimeric mice grafted with human astrocytes, which exhibit 3 fold larger and faster Ca^2+^ signals, display greatly enhanced plasticity and learning (Han et al., 2013), supporting the notion that in contrast with the “all or nothing” action potential system utilized by neurons, for Ca^2+^ astroglial signaling, size matters. One study reported in culture that bath application for 5 min of 100 μM and 1 mM of L-glutamate increases astrocytic Ca^2+^ concentration by ~400 and ~800 nM, respectively (Floyd et al., 2005). These uniform and long lasting elevated concentrations of glutamate are likely to be non-physiological, although other work on cultured astrocytes has shown that Ca^2+^ wave propagation results in accumulation of glutamate in the range of 1–100 μM (Innocenti et al., 2000). Further, Parpura and Haydon reported that more physiological increases in Ca^2+^ levels, from 84 to 140 nM, induced by flash photolysis, is sufficient to trigger SICs, proposed to be mediated by astroglial glutamate release (Parpura and Haydon, 2000). These investigations were, however, performed on cultured cells with artificial astroglial stimulations. Astroglial intracellular Ca^2+^ levels that actually occur in response to basal synaptic transmission and plasticity *in situ* therefore remain unknown.

Corroborating hippocampal investigations, slow neuronal depolarizations recorded in pyramidal neurons from mouse visual cortex slices and induced by brief (0.2–1 s) pulse application of acetylcholine through a glass pipette is also drastically reduced by prior BAPTA infusion *via* a patch pipette in the astrocytic network, as acetylcholine evokes a Ca^2+^ rise in astrocytes of this brain region. Since such neuronal depolarization was shown to rely on NMDA receptor activation, the authors suggest that astrocytic activation may lead to glutamate or D-serine gliotransmission, or to Ca^2+^ dependent regulation of extracellular K^+^ and/or glutamate (Chen et al., 2012). With regard to the astroglial signaling subtending these regulations involving gliotransmission, an outstanding investigation aiming at deciphering the mechanism of astrocytic glutamate release used dual patch clamp to elegantly measure the actual release of glutamate from cultured astrocytes. The trick used in this study was to patch a HEK293T “sniffer cell” expressing a non-desensitizing form of AMPA receptors and placed in direct apposition to an astrocyte (Figures 5C–E). The latter is then activated either by application of TFLLR, a selective peptide agonist of the PAR-1 G-protein coupled receptor, or by patching the cell using an intra-pipette solution containing high Ca^2+^ concentrations. After screening the different pathways that might be involved in glutamate release, the authors conclude that a fast and slow mode of astrocytic glutamate release co-exist and are mediated by glutamate permeable two-pore domain K^+^ channel TREK-1 and glutamate permeable Ca^2+^ activated anion channel Best1, respectively (Woo et al., 2012).

In sharp contrast to the aforementioned up-regulatory action of astrocytic glutamate on neuronal activity, Ca^2+^ chelation in barrel cortex astrocytes has been found to increase the frequency of spontaneous excitatory postsynaptic potentials, as well as evoked neuronal depolarization and excitability recorded in patch clamp conditions, suggesting in this case a down-regulatory role of neuroglial signaling. Although the precise mechanism could not be completely unraveled, it appeared to involve GABAergic signaling suggesting that, in this brain region, GABAergic gliotransmission could bridle neuronal activity (Benedetti et al., 2011). This finding is in line with the demonstration, in the mouse olfactory bulb, that both slow inward (SIC, excitatory) and outward (SOC, inhibitory) currents are observed spontaneously, can be evoked by mechanical stimulation of astrocytes and are attributable to the glial release of glutamate and GABA, respectively (Kozlov et al., 2006). Down-regulation of synaptic activity as a result of glial cell stimulation has also been demonstrated in the cerebellum, where directly applied depolarizing steps on patch clamped astrocytes in the imposed voltage configuration resulted in a marked decrease in the frequency of Purkinje neurons spontaneous postsynaptic potentials (Brockhaus and Deitmer, 2002). Finally, the inhibitory action of astrocytes on neuronal activation has also been shown in CA1 neurons to include the participation of local circuit interneurons, since an astrocytic Ca^2+^ rise dependent on GABA_B_ receptor activation was found to increase the frequency of inhibitory postsynaptic currents, an effect blocked by intra-astrocytic application of BAPTA (Kang et al., 1998). Altogether, this body of investigations indicates that astrocytic Ca^2+^ activation leads to the release of neuroactive molecules able to up- as well as down-regulate synaptic and neuronal activity.

The importance of such form of “astrocytic excitability” is empowered by the fact that extracellular pathways involving gliotransmitters such as ATP, and potentially amplification of signaling through gap junction mediated astroglial networks, likely contribute to the propagation of intercellular Ca^2+^ waves or glissandi, as recently reported *in vivo* (Kuga et al., 2011). This strongly suggests an important role for astrocytes, at the individual cell or network level, in the synchronization of neuronal activities (Cornell-Bell et al., 1990; Dani et al., 1992; Angulo et al., 2004; Fellin et al., 2004; Wang et al., 2006). Yet, this is subject to controversies as recent data suggest, on the contrary, that specific Ca^2+^ activation does not influence neurotransmission (Fiacco et al., 2007; Agulhon et al., 2010). In these investigations, specific Ca^2+^ activation was achieved through the use of transgenic mice in which astrocytes express MrgA1 Gq-coupled receptors that respond to a specific agonist and are normally not found in the CNS. Limitations of this approach, however, include the fact that uniform Ca^2+^ increase induced by activation of a receptor that is exotic to the CNS may not be linked to intracellular signaling that is relevant in terms of gliotransmission. Besides, expression of receptors leading to the release of neuroactive molecules is expected to be targeted to specific cellular locations, which is not the case of this non-physiological expression MrgA1 receptors. The same research group also demonstrates that obliterating Ca^2+^ activation in a mouse line in which astrocytic inositol triphosphate receptors (i.e., InsP3R2) are knocked out also results in unaltered basal transmission, short-, and long-term plasticity, indicating that Ca^2+^ release from glial intracellular stores is not a *sine qua non* condition to normal neuronal activity (Petravicz et al., 2008; Agulhon et al., 2010). However, InsP3-dependent source of astroglial Ca^2+^ may not be at play in the regulation of synaptic transmission and plasticity.

#### Sodium signaling and energy metabolism

In addition, despite overwhelming evidence pointing toward the key function of Ca^2+^ elevation in astrocytes, one should bear in mind that the latter is to be considered as one facet of the multiple mechanisms at play in the astroglial control of synaptic transmission. Accordingly, using patch clamp recording of neurons and astrocytes coupled to Ca^2+^ imaging, an early investigation performed on cortical primary culture unraveled rapid inward glial currents that coincided with bursts of electrical activity in neighboring neurons, and which were not associated with Ca^2+^ signal; the authors further stressed the fact that the temporal scale of the slow Ca^2+^ waves does not match the fast astroglial current observed, more reminiscent of a depolarization due to AMPA receptor activation or glutamate transport (Murphy and Wier, 1993). Recent investigations combining intracellular recordings with sodium (Na^+^) imaging, utilizing the fluorescent Na^+^ indicator SBFI, showed in cerebellar Bergmann glial cells that bursts of activity rapidly triggers Na^+^ signals attributable to AMPA receptor activation, as well as Na^+^ dependent glutamate uptake (Kirischuk et al., 2007; Bennay et al., 2008). Furthermore, Na^+^ rise in astroglia in response to neuronal activity has been found in cultures and in hippocampal slices to result in propagating Na^+^ waves (Bernardinelli et al., 2004; Langer et al., 2012). Interestingly, although the work performed on cultured astrocytes suggested that Ca^2+^ and Na^+^ waves are intrinsically linked, as chelation of Ca^2+^ also resulted in Na^+^ wave abolition, the recent investigation performed *in situ* demonstrates that Ca^2+^ signaling up-regulates, but is not a prerequisite for the dispersion of Na^+^ through the astroglial network (Langer et al., 2012). The impact of interfering with this phenomenon on neuronal activity has been difficult to investigate, as no way of specifically chelating Na^+^ in astrocytes is currently available. Developing such tool would thus be greatly beneficial to our understanding of neuroglial interactions.

Yet, such increases in Na^+^ concentrations have been shown to activate the Na^+^/K^+^ ATPase, resulting in a high energy demand and consequently to an increased glucose uptake though the glucose transporter GLUT1, thereby initiating the astrocyte-neuron lactate shuttle consisting in the activation of phosphoglycerate kinases that triggers glycolysis and thus results in the production of lactate, released in the extracellular space and taken up by neurons (Pellerin and Magistretti, 2012). Na^+^ waves are therefore thought to translate into metabolic waves (Pellerin and Magistretti, 1994; Chatton et al., 2000, 2003; Bernardinelli et al., 2004). Thus, through the generation of ionic waves in the astrocytic net, local neuronal activity increases astrocyte to neuron lactate supply toward *loci* of high energy demand. Strikingly, we were able to directly show such activity-dependent preferential supply of energy metabolites mediated by gap junctional inter-astrocytic communication, using the fluorescent glucose analog 2-NBDG (Rouach et al., 2008). Indeed, by experimentally creating a high local energy demand in the *stratum radiatum* (using 1 Hz electrical stimulation) and observing the 2-NBDG diffusion from a single astrocyte patched in the *stratum oriens*, it was found that the fluorescent metabolite spread through the astroglial network is extended and directed toward the site of high neuronal activity. Further, the efficiency of such astrocytic metabolic supply was demonstrated by recovering the loss of neurotransmission normally observed in glucose deprivation conditions, *via* selective application of glucose in the astroglial network (Rouach et al., 2008).

#### Potassium signaling

Activation of Na^+^/K^+^ ATPase by intracellular Na^+^ rise also implies a concomitant K^+^ entry occurring as a result of extruding Na^+^. Uptake of K^+^ in astrocytes is also known to strongly depend on inwardly rectifying K^+^ channels, notably K_ir_4.1 (Olsen and Sontheimer, 2008). K_ir_ channels can, however, transport K^+^ out or inside the cell depending on the K^+^ electrochemical gradient, and have been reported to mainly undertake maintenance of the pronounced astrocytic resting membrane potential, and to be only moderately involved in extracellular K^+^ clearance as compared to the Na^+^/K^+^ ATPase (Ransom et al., 2000; Xiong and Stringer, 2000; D'Ambrosio et al., 2002). D'Ambrosio and colleagues claimed that pharmacological blockade of K_ir_ channels with bath application of barium does increase basal concentration of extracellular K^+^, but leaves intact its clearance after high frequency neuronal stimulation; instead, such clearance is heavily disturbed by inhibition of the Na^+^/K^+^ ATPase using ouabain (D'Ambrosio et al., 2002). While barium impact on K^+^ uptake is still controversial (Xiong and Stringer, 2000; Jauch et al., 2002; Meeks and Mennerick, 2007), the effect of ouabain is consistent among studies (Förstl et al., 1982; Ransom et al., 2000). However, these findings suffer from the pharmacological tools used, which lack astrocytic specificity, and most likely disturb general ionic homeostasis as the main role of Na^+^/K^+^ ATPase is to maintain transmembrane ionic gradients. Further, these results have been contradicted by a recent *ex vivo* study on hippocampal slices from K_ir_4.1 knockout mice (Haj-Yasein et al., 2011). This also reinforces previous investigations showing that K_ir_4.1 channels promote K^+^ uptake after moderate extracellular rises (Neusch et al., 2006; Chever et al., 2010). Together, these studies indicate that both Na^+^/K^+^ ATPases and astroglial K_ir_ channels are implicated in K^+^ excess uptake. These mechanisms therefore work synergically in maintaining K^+^ homeostasis. In particular Na^+^/K^+^ ATPase activity could lead to membrane hyperpolarization, thereby favoring K^+^ entry through K^+^ channels. The relative contribution of Na^+^/K^+^ ATPases and K_ir_ channels most probably vary according to the regime of activity (Somjen et al., 2008). Na^+^/K^+^ ATPases efficiency is indeed prominent in case of strong neuronal activity. In this context, the main current view actually proposes that K_ir_4.1 channels could also be responsible for counterbalancing K^+^ uptake through Na^+^/K^+^ ATPase activity (D'Ambrosio et al., 2002; Haj-Yasein et al., 2011; Bay and Butt, 2012; Bay and Butt, but see Chever et al., 2010).

Noteworthy, the pharmacological and genetic tools used to study Na^+^/K^+^ ATPases and K_ir_ functions in astrocytes lack specificity and target different cell types, such as neurons or oligodendrocytes. One acute way of accurately invalidating K_ir_ function in astrocytes would be to apply barium intracellularly in the astroglial network through a patch pipette, as this ion is a potent K_ir_ channel blocker inside, as well as outside the cell (Solessio et al., 2000). Alternatively, delivery of K_ir_ antibodies through the patch electrode has been shown to be an efficient blocker in bipolar cells of the retina (Raz-prag et al., 2010), and might therefore represent an interesting way of specifically assess K_ir_ functions in astrocytes. However, such approach might not be efficient in all cell types, as in our hands, intrapipette application of K_ir_ antibodies in hippocampal astrocytes resulted in no alteration of neither glial nor neuronal currents (Sibille and Rouach, unpublished observations).

In sum, blocking particular sets of astrocytic functions and recording the consequences on neuronal activity and/or on regulation of extracellular medium composition has proven to be a very informative approach to explore the relevance of neuroglial interactions and has enabled to unravel the importance of gliotransmission, metabolic coupling and ionic wave propagation in the regulation of neuronal activity. Yet, the relatively limited number of tools allowing disruption of specific functions in comparison with the tremendous number of elements involved in neuroglial signaling has, to some extent, biased this field of research toward mechanisms that are possible to assess, thereby preventing a clear discernment of the whole picture sketching out. In particular, one blatant example is the direct correspondence often used between astrocytic activation and Ca^2+^ signaling, which is most certainly attributable to the availability of chelators that are specific to Ca^2+^ as opposed, for instance, to Na^+^ or K^+^.

## Summary and conclusions: what electrophysiology can and cannot assess in neuroglial interactions

Excitability of astrocytes has been proposed to lie primarily in their dynamic Ca^2+^ signaling, rather than their electrical responses. Thus, in the last decades, to unravel the neuroglial dialog engaged in processing brain information, the main focus of physiologists from the glia field has been Ca^2+^ signaling using imaging at the levels of astroglial microdomain, single cells and networks. This body of work has provided a wealth of valuable information which has tremendously advanced our understanding of neuroglial interactions. However, signaling in astrocytes also includes other ionic players. Indeed, membrane depolarization induced by neuronal activity was the first activity dependent signal identified in glia (Orkand et al., 1966). Since then, astroglial ionic responses other than Ca^2+^ received less attention because of their slow time scale, the passive membrane properties of glia, and the lack of selective tools to assess their functional consequences. However, recent data have revealed that astrocytes express on their membrane a variety of ion channels, transmitter receptors and transporters, which mediate alternative signaling pathways, via for instance Na^+^. In addition, molecular tools targeting specific glial ionic channels have been developed. Thus, the simplistic view of astrocytes as passive cells that express only leak K^+^ channels undergoing passive membrane potential fluctuations needs to be updated. The information reviewed herein show that electrophysiology is a valuable online technique, which has provided major insights on the dynamic neuroglial ionic dialogue mediating information processing at the cellular and molecular level. In particular, dual recordings of synaptically evoked neuronal and astroglial responses have generated information about concomitant alterations in the activity of pre- or postsynaptic elements and associated astrocytes. Thanks to their ionic signaling, astrocytes are now promoted to both, good electrophysiological readouts and important regulators of synaptic activity (Diamond et al., 1998; Lüscher et al., 1998; Djukic et al., 2007; Henneberger and Rusakov, 2012; Pannasch et al., 2012b). The astroglial depolarization evoked synaptically is a direct measure of the increase in extracellular K^+^ levels (Amzica, 2002), occurring as a result of presynaptic action potential firing and subsequent postsynaptic depolarization (Poolos et al., 1987). Thus, astroglial membrane potential dynamics is a good sensor for changes in presynaptic excitability, postsynaptic activity, extracellular space volume and K^+^ buffering capacities (Amzica, 2002; Pannasch et al., 2012a). Alternatively, the GLT current from astrocytes is a reliable detector of glutamate release from presynaptic terminals, and can thus monitors short-term changes in release probability (Bergles and Jahr, 1997; Diamond et al., 1998; Lüscher et al., 1998). Such transporter currents may also reflect the level of astroglial synapse coverage, which is known to be plastic during various physiopathological conditions (Wenzel et al., 1991; Hawrylak et al., 1993; Genoud et al., 2006; Lushnikova et al., 2009; Oliet and Bonfardin, 2010), and are thus good indicators of morphological and functional neuroglial interactions. But astroglial membrane potential dynamics and GLT currents do not only reflect synaptic activity. They are also regulated during development and in numbers of physiopathological conditions, including by neuronal activity or epilepsy, *via* changes in the expression, localization and function of K_ir_ channels and GLT, respectively, and can thus directly affect neighboring synaptic activity and plasticity (Djukic et al., 2007; Tzingounis and Wadiche, 2007; Jabs et al., 2008; Benediktsson et al., 2012). Therefore, combining electron microscopy, biochemistry or imaging to electrophysiolology is now crucial to decipher whether ionic changes detected on astroglial membranes just reflect, or rather cause, alterations in neuronal activity.

## Limits and perspectives

Up to now, electrophysiological whole cell recordings from astrocytes are primarily performed at the level of the soma. Such recordings allow detection of currents which mostly originate from the cell soma or proximal processes, and whose identity is still unclear. Somatic passive conductances are thought to be responsible for the major leak of currents occurring in response to somatic current injections applied to perform IV curves. These passive currents likely subtend the typical low membrane resistance of astrocytes. Determining the components of somatic passive conductances is therefore a major issue to reliably assess the electrophysiology of mature astrocytes, especially in tissues. K2P (Seifert et al., 2009; Zhou et al., 2009) and gap junction channels (Seifert et al., 2009; Olsen, 2012) have been proposed to contribute to passive currents, although the involvement of connexin channels is controversial (Schools et al., 2006; Wallraff et al., 2006; Pannasch et al., 2011). Identifying the nature of somatic passive currents would open the possibility of deterring such major conductances, hence increasing the astroglial membrane resistance, and thereby unmask small non-passive activity dependent conductances that may be involved in sensing and modulating synaptic activity. In addition, novel pharmacological or genetic tools are also needed to unravel the role of non-passive conductances in astroglial physiopathology and neuronal activity.

Thus, basal activity of channels and receptors in fine distal perisynaptic astrocyte processes (PAPs) is currently hardly detectable, due in part to the low spatial and temporal control of membrane currents and potentials by patch clamp recordings of astrocytes *in situ* (Zhou et al., 2009). Such limit is unfortunate, because the surface of the tiny astroglial processes exceeds by far the membrane area of the soma and main processes, and the PAPs are the most interesting *loci* with regard to astroglial regulation of neurotransmission as they contain the functionally relevant channels, transporters and receptors, such as K_ir_4.1 channels and GLT, which are likely the crucial players in neuroglial interactions and synaptic modulation. Therefore, dual patch clamp recording of astrocytes and neurons cannot be used to study the dialog between individual synapses and neighboring fine astroglial processes occurring during basal spontaneous activity. Instead, such recordings may be useful to investigate the integration by astrocytes of coordinated activity from neuronal assemblies occurring particularly during afference stimulation. It is thus no wonder that isolation of synaptically activated currents in astrocytes, such as GLT currents, is delicate; it requires the use of pharmacology, to inhibit the main K^+^ conductances and it is also often necessary to boost synaptic activity in order to increase the observable astroglial response. However, such manipulations results in experimental conditions drifting away from the physiological situation and may thus not relate to the native neuroglial dialog occurring in basal conditions. In addition, the actual time course of astroglial glutamate clearance derived from the recorded GLT current can be partially obscured by current filtering, which distorts their kinetics, due to diverse factors such as astroglial electrotonic properties and asynchronous transmitter release. Nevertheless, methods utilized to extract the temporal features of the filtering mechanisms can be used to derive the actual glutamate clearance time course in physiological or pathological situations, as recently performed (Diamond, 2005; Scimemi et al., 2009; Pannasch et al., 2011).

Ideally, to investigate local astroglial ionic currents triggered by basal synaptic transmission, patch clamp recordings from fine astroglial processes should be developed, in a similar fashion to what is now currently performed on dendrites (Davie et al., 2006). Although patching fine perisynaptic astroglial processes will most likely be challenging because of their tiny size, it would permit to decipher the intimate communication ongoing between astroglial microdomains and individual synapses. However, to be detected, electrophysiological responses recorded from individual astroglial processes would need to display an amplitude above threshold detection (~5 pA), because electrical noise can reach ~2–4 pA in patch clamp recordings. Alternatively, the use of voltage sensitive dyes could reveal the heterogeneity of membrane potentials in astrocytes, and help defining whether fine perisynaptic astroglial processes play active functions mediated by K^+^ channels, enriched in such processes. Yet, dual electrophysiological recording from astrocytes and neurons offers quantitative information about all ionic currents, and thus strikes as being a unique and efficient method to dissect online the dynamics of neuroglial ionic signaling and its role in information processing.

### Conflict of interest statement

The authors declare that the research was conducted in the absence of any commercial or financial relationships that could be construed as a potential conflict of interest.

## References

[B1] Adermark L. Lovinger D. M. (2008). Electrophysiological properties and gap junction coupling of striatal astrocytes. Neurochem. Int. 52, 1365–1372 10.1016/j.neuint.2008.02.006 18396351 PMC2442918

[B2] Agulhon C. Fiacco T. A. McCarthy K. D. (2010). Hippocampal short- and long-term plasticity are not modulated by astrocyte Ca2+ signaling. Science 327, 1250–1254 10.1126/science.1184821 20203048

[B3] Aitken P. G. Somjen G. G. (1986). The sources of extracellular potassium accumulation in the CA1 region of hippocampal slices. Brain Res. 369, 163–167 10.1016/0006-8993(86)90524-X 3697739

[B4] Amato A. Barbour B. Szatkowski M. Attwell D. (1994). Counter-transport of potassium by the glutamate uptake carrier in glial cells isolated from the tiger salamander retina. J. Physiol. 479, 371–380 7837095 10.1113/jphysiol.1994.sp020302 PMC1155756

[B5] Amedee T. Robert A. Coles J. A. (1997). Potassium homeostasis and glial energy metabolism. Glia 21, 46–55 10.1002/(SICI)1098-1136(199709)21:1<46::AID-GLIA5>3.0.CO;2-# 9298846

[B6] Amzica F. (2002). *In vivo* electrophysiological evidences for cortical neuron–glia interactions during slow (<1 Hz) and paroxysmal sleep oscillations. J. Physiol. Paris 96, 209–219 10.1016/S0928-4257(02)00008-6 12445898

[B7] Amzica F. Massimini M. (2002). Glial and neuronal interactions during slow wave and paroxysmal activities in the neocortex. Cereb. Cortex 12, 1101–1113 10.1093/cercor/12.10.1101 12217974

[B8] Amzica F. Massimini M. Manfridi A. (2002). Spatial buffering during slow and paroxysmal sleep oscillations in cortical networks of glial cells *in vivo*. J. Neurosci. 22, 1042–1053 11826133 10.1523/JNEUROSCI.22-03-01042.2002 PMC6758489

[B9] Amzica F. Neckelmann D. (1999). Membrane capacitance of cortical neurons and glia during sleep oscillations and spike-wave seizures. J. Neurophysiol. 82, 2731–2746 10561441 10.1152/jn.1999.82.5.2731

[B10] Anderson C. M. Swanson R. A. (2000). Astrocyte glutamate transport: review of properties, regulation, and physiological functions. Glia 32, 1–14 10.1002/1098-1136(200010)32:1<1::AID-GLIA10>3.3.CO;2-N 10975906

[B11] Angulo M. C. Kozlov A. S. Charpak S. Audinat E. (2004). Glutamate released from glial cells synchronizes neuronal activity in the hippocampus. J. Neurosci. 24, 6920–6927 10.1523/JNEUROSCI.0473-04.2004 15295027 PMC6729611

[B12] Araque A. Martín E. D. Perea G. Arellano J. I. Buño W. (2002). Synaptically released acetylcholine evokes Ca2+ elevations in astrocytes in hippocampal slices. J. Neurosci. 22, 2443–2450 11923408 10.1523/JNEUROSCI.22-07-02443.2002 PMC6758296

[B13] Araque A. Parpura V. Sanzgiri R. P. Haydon P. G. (1998a). Glutamate-dependent astrocyte modulation of synaptic transmission between cultured hippocampal neurons. Eur. J. Neurosci. 10, 2129–2142 10.1046/j.1460-9568.1998.00221.x 9753099

[B14] Araque A. Sanzgiri R. P. Parpura V. Haydon P. G. (1998b). Calcium elevation in astrocytes causes an NMDA receptor-dependent increase in the frequency of miniature synaptic currents in cultured hippocampal neurons. J. Neurosci. 18, 6822–6829 9712653 10.1523/JNEUROSCI.18-17-06822.1998 PMC6792963

[B15] Ballanyi K. Grafe P. ten Bruggencate G. (1987). Ion activities and potassium uptake mechanisms of glial cells in guinea-pig olfactory cortex slices. J. Physiol. 382, 159–174 2442359 10.1113/jphysiol.1987.sp016361 PMC1183018

[B16] Barbour B. Brew H. Attwell D. (1988). Electrogenic glutamate uptake in glial cells is activated by intracellular potassium. Nature 335, 433–435 10.1038/335433a0 2901670

[B17] Barbour B. Brew H. Attwell D. (1991). Electrogenic uptake of glutamate and aspartate into glial cells isolated from the salamander (Ambystoma) retina. J. Physiol. 436, 169–193 1676418 10.1113/jphysiol.1991.sp018545 PMC1181500

[B18] Barres B. A. (1991). Glial ion channels. Curr. Opin. Neurobiol. 1, 354–359 10.1016/0959-4388(91)90052-9 1726551

[B19] Bay V. Butt A. M. (2012). Relationship between glial potassium regulation and axon excitability: a role for glial Kir4.1 channels. Glia 60, 651–660 10.1002/glia.22299 22290828

[B20] Bellamy T. C. Ogden D. (2005). Short-term plasticity of Bergmann glial cell extrasynaptic currents during parallel fiber stimulation in rat cerebellum. Glia 52, 325–335 10.1002/glia.20248 16078233

[B21] Bellamy T. C. Ogden D. (2006). Long-term depression of neuron to glial signalling in rat cerebellar cortex. Eur. J. Neurosci. 23, 581–586 10.1111/j.1460-9568.2005.04588.x 16420466

[B22] Ben Achour S. Pascual O. (2012). Astrocyte-neuron communication: functional consequences. Neurochem. Res. 37, 2464–2473 10.1007/s11064-012-0807-0 22669630

[B23] Benedetti B. Matyash V. Kettenmann H. (2011). Astrocytes control GABAergic inhibition of neurons in the mouse barrel cortex. J. Physiol. 589, 1159–1172 10.1113/jphysiol.2010.203224 21224221 PMC3060594

[B24] Benediktsson A. M. Marrs G. S. Tu J. C. Worley P. F. Rothstein J. D. Bergles D. E. (2012). Neuronal activity regulates glutamate transporter dynamics in developing astrocytes. Glia 60, 175–188 10.1002/glia.21249 22052455 PMC3232333

[B25] Bennay M. Langer J. Meier S. D. Kafitz K. W. Rose C. R. (2008). Sodium signals in cerebellar Purkinje neurons and Bergmann glial cells evoked by glutamatergic synaptic transmission. Glia 56, 1138–1149 10.1002/glia.20685 18442095

[B26] Bergles D. E. Jabs R. Steinhauser C. (2010). Neuron-glia synapses in the brain. Brain Res. Rev. 63, 130–137 10.1016/j.brainresrev.2009.12.003 20018210 PMC2862892

[B27] Bergles D. E. Jahr C. E. (1997). Synaptic activation of glutamate transporters in hippocampal astrocytes. Neuron 19, 1297–1308 10.1016/S0896-6273(00)80420-1 9427252

[B28] Bernardinelli Y. Chatton J.-Y. (2008). Differential effects of glutamate transporter inhibitors on the global electrophysiological response of astrocytes to neuronal stimulation. Brain Res. 1240, 47–53 10.1016/j.brainres.2008.09.014 18823961

[B29] Bernardinelli Y. Magistretti P. J. Chatton J.-Y. (2004). Astrocytes generate Na+-mediated metabolic waves. Proc. Natl. Acad. Sci. U.S.A. 101, 14937–14942 10.1073/pnas.0405315101 15466714 PMC522032

[B30] Bordey A. Lyons S. A. Hablitz J. J. Sontheimer H. (2001). Electrophysiological characteristics of reactive astrocytes in experimental cortical dysplasia. J. Neurophysiol. 85, 1719–1731 11287494 10.1152/jn.2001.85.4.1719

[B31] Bordey A. Sontheimer H. (1997). Postnatal development of ionic currents in rat hippocampal astrocytes *in situ*. J. Neurophysiol. 78, 461–477 9242294 10.1152/jn.1997.78.1.461

[B32] Bordey A. Sontheimer H. (1998). Electrophysiological properties of human astrocytic tumor cells *in situ*: enigma of spiking glial cells. J. Neurophysiol. 79, 2782–2793 9582244 10.1152/jn.1998.79.5.2782

[B33] Brew H. Attwell D. (1987). Electrogenic glutamate uptake is a major current carrier in the membrane of axolotl retinal glial cells. Nature 327, 707–709 10.1038/327707a0 2885752

[B34] Brockhaus J. Deitmer J. W. (2002). Long-lasting modulation of synaptic input to Purkinje neurons by Bergmann glia stimulation in rat brain slices. J. Physiol. 545, 581–593 10.1113/jphysiol.2002.028423 12456836 PMC2290679

[B35] Butt A. M. Kalsi A. (2006). Inwardly rectifying potassium channels (Kir) in central nervous system glia: a special role for Kir4.1 in glial functions. J. Cell. Mol. Med. 10, 33–44 10.1111/j.1582-4934.2006.tb00289.x 16563220 PMC3933100

[B36] Castellucci V. F. Goldring S. (1970). Contribution to steady potential shifts of slow depolarization in cells presumed to be glia. Electroencephalogr. Clin. Neurophysiol. 28, 109–118 10.1016/0013-4694(70)90178-1 4189522

[B37] Chatton J. Y. Marquet P. Magistretti P. J. (2000). A quantitative analysis of L-glutamate-regulated Na+ dynamics in mouse cortical astrocytes: implications for cellular bioenergetics. Eur. J. Neurosci. 12, 3843–3853 10.1046/j.1460-9568.2000.00269.x 11069579

[B38] Chatton J.-Y. Pellerin L. Magistretti P. J. (2003). GABA uptake into astrocytes is not associated with significant metabolic cost: implications for brain imaging of inhibitory transmission. Proc. Natl. Acad. Sci. U.S.A. 100, 12456–12461 10.1073/pnas.2132096100 14530410 PMC218779

[B39] Chaudhry F. A. Lehre K. P. Lookeren Campagne M. van, Ottersen O. P. Danbolt N. C. Storm-Mathisen J. (1995). Glutamate transporters in glial plasma membranes: highly differentiated localizations revealed by quantitative ultrastructural immunocytochemistry. Neuron 15, 711–720 10.1016/0896-6273(95)90158-2 7546749

[B40] Chen N. Sugihara H. Sharma J. Perea G. Petravicz J. Le C. (2012). Nucleus basalis-enabled stimulus-specific plasticity in the visual cortex is mediated by astrocytes. Proc. Natl. Acad. Sci. U.S.A. 109, E2832–E2841 10.1073/pnas.1206557109 23012414 PMC3478642

[B41] Chesler M. Kraig R. P. (1987). Intracellular pH of astrocytes increases rapidly with cortical stimulation. Am. J. Physiol. 253, R666–R670 3116863 10.1152/ajpregu.1987.253.4.R666 PMC2805720

[B42] Chesler M. Kraig R. P. (1989). Intracellular pH transients of mammalian astrocytes. J. Neurosci. 9, 2011–2019 2723764 10.1523/JNEUROSCI.09-06-02011.1989 PMC2690820

[B43] Chever O. Djukic B. McCarthy K. D. Amzica F. (2010). Implication of Kir4.1 channel in excess potassium clearance: an *in vivo* study on anesthetized glial-conditional Kir4.1 knock-out mice. J. Neurosci. 30, 15769–15777 10.1523/JNEUROSCI.2078-10.2010 21106816 PMC6633770

[B44] Chvátal A. Pastor A. Mauch M. Syková E. Kettenmann H. (1995). Distinct populations of identified glial cells in the developing rat spinal cord slice: ion channel properties and cell morphology. Eur. J. Neurosci. 7, 129–142 10.1111/j.1460-9568.1995.tb01027.x 7536092

[B45] Clark B. A. Barbour B. (1997). Currents evoked in Bergmann glial cells by parallel fibre stimulation in rat cerebellar slices. J. Physiol. 502, 335–350 10.1111/j.1469-7793.1997.335bk.x 9263914 PMC1159553

[B46] Connors B. W. Ransom B. R. Kunis D. M. Gutnick M. J. (1982). Activity-dependent K+ accumulation in the developing rat optic nerve. Science 216, 1341–1343 10.1126/science.7079771 7079771

[B47] Cornell-Bell A. H. Finkbeiner S. M. Cooper M. S. Smith S. J. (1990). Glutamate induces calcium waves in cultured astrocytes: long-range glial signaling. Science 247, 470–473 10.1126/science.1967852 1967852

[B48] D'Ambrosio R. Gordon D. S. Winn H. R. (2002). Differential role of KIR channel and Na(+)/K(+)-pump in the regulation of extracellular K(+) in rat hippocampus. J. Neurophysiol. 87, 87–102 11784732 10.1152/jn.00240.2001

[B49] Danbolt N. C. (2001). Glutamate uptake. Prog. Neurobiol. 65, 1–105 10.1016/S0301-0082(00)00067-8 11369436

[B50] Dani J. W. Chernjavsky A. Smith S. J. (1992). Neuronal activity triggers calcium waves in hippocampal astrocyte networks. Neuron 8, 429–440 10.1016/0896-6273(92)90271-E 1347996

[B51] Davie J. T. Kole M. H. P. Letzkus J. J. Rancz E. A. Spruston N. Stuart G. J. (2006). Dendritic patch-clamp recording. Nat. Protoc. 1, 1235–1247 10.1038/nprot.2006.164 17406407 PMC7616975

[B52] Deitmer J. W. Rose C. R. (2010). Ion changes and signalling in perisynaptic glia. Brain Res. Rev. 63, 113–129 10.1016/j.brainresrev.2009.10.006 19895844

[B53] De Pina-Benabou M. H. Srinivas M. Spray D. C. Scemes E. (2001). Calmodulin kinase pathway mediates the K+-induced increase in Gap junctional communication between mouse spinal cord astrocytes. J. Neurosci. 21, 6635–6643 11517253 10.1523/JNEUROSCI.21-17-06635.2001 PMC1513544

[B167] De Saint Jan D. (2005). Detecting activity in olfactory bulb glomeruli with astrocyte recording. J. Neurosci. 25, 2917–2924 10.1523/JNEUROSCI.5042-04.2005 15772351 PMC6725148

[B54] Di Castro M. A. Chuquet J. Liaudet N. Bhaukaurally K. Santello M. Bouvier D. (2011). Local Ca2+ detection and modulation of synaptic release by astrocytes. Nat. Neurosci. 14, 1276–1284 10.1038/nn.2929 21909085

[B55] Diamond J. S. (2005). Deriving the glutamate clearance time course from transporter currents in CA1 hippocampal astrocytes: transmitter uptake gets faster during development. J. Neurosci. 25, 2906–2916 10.1523/JNEUROSCI.5125-04.2005 15772350 PMC6725141

[B56] Diamond J. S. Bergles D. E. Jahr C. E. (1998). Glutamate release monitored with astrocyte transporter currents during LTP. Neuron 21, 425–433 10.1016/S0896-6273(00)80551-6 9728923

[B57] Djukic B. Casper K. B. Philpot B. D. Chin L.-S. McCarthy K. D. (2007). Conditional knock-out of Kir4.1 leads to glial membrane depolarization, inhibition of potassium and glutamate uptake, and enhanced short-term synaptic potentiation. J. Neurosci. 27, 11354–11365 10.1523/JNEUROSCI.0723-07.2007 17942730 PMC6673037

[B58] Dufour S. Dufour P. Chever O. Vallée R. Amzica F. (2011). *In vivo* simultaneous intra- and extracellular potassium recordings using a micro-optrode. J. Neurosci. Methods 194, 206–217 10.1016/j.jneumeth.2010.10.004 20951737

[B59] Enkvist M. O. McCarthy K. D. (1994). Astroglial gap junction communication is increased by treatment with either glutamate or high K+ concentration. J. Neurochem. 62, 489–495 10.1046/j.1471-4159.1994.62020489.x 7905024

[B60] Fellin T. Pascual O. Gobbo S. Pozzan T. Haydon P. G. Carmignoto G. (2004). Neuronal synchrony mediated by astrocytic glutamate through activation of extrasynaptic NMDA receptors. Neuron 43, 729–743 10.1016/j.neuron.2004.08.011 15339653

[B61] Fiacco T. A. Agulhon C. Taves S. R. Petravicz J. Casper K. B. Dong X. (2007). Selective stimulation of astrocyte calcium *in situ* does not affect neuronal excitatory synaptic activity. Neuron 54, 611–626 10.1016/j.neuron.2007.04.032 17521573

[B62] Fiacco T. A. McCarthy K. D. (2004). Intracellular astrocyte calcium waves *in situ* increase the frequency of spontaneous AMPA receptor currents in CA1 pyramidal neurons. J. Neurosci. 24, 722–732 10.1523/JNEUROSCI.2859-03.2004 14736858 PMC6729258

[B63] Floyd C. L. Gorin F. A. Lyeth B. G. (2005). Mechanical strain injury increases intracellular sodium and reverses Na+/Ca2+ exchange in cortical astrocytes. Glia 51, 35–46 10.1002/glia.20183 15779085 PMC2996279

[B64] Förstl J. Galvan M. ten Bruggencate G. (1982). Extracellular K+ concentration during electrical stimulation of rat isolated sympathetic ganglia, vagus and optic nerves. Neuroscience 7, 3221–3229 10.1016/0306-4522(82)90244-5 7162635

[B65] Gabriel S. Kivi A. Eilers A. Kovács R. Heinemann U. (1998). Effects of barium on stimulus-induced rises in [K+]o in juvenile rat hippocampal area CA1. Neuroreport 9, 2583–2587 10.1097/00001756-199808030-00029 9721937

[B66] Ge W.-P. Duan S. (2007). Persistent enhancement of neuron-glia signaling mediated by increased extracellular K+ accompanying long-term synaptic potentiation. J. Neurophysiol. 97, 2564–2569 10.1152/jn.00146.2006 17035364

[B67] Genoud C. Quairiaux C. Steiner P. Hirling H. Welker E. Knott G. W. (2006). Plasticity of astrocytic coverage and glutamate transporter expression in adult mouse cortex. PLoS Biol. 4:e343 10.1371/journal.pbio.0040343 17048987 PMC1609127

[B68] Giaume C. Fromaget C. el Aoumari A. Cordier J. Glowinski J. Gros D. (1991). Gap junctions in cultured astrocytes: single-channel currents and characterization of channel-forming protein. Neuron 6, 133–143 10.1016/0896-6273(91)90128-M 1702648

[B69] Giaume C. Koulakoff A. Roux L. Holcman D. Rouach N. (2010). Astroglial networks: a step further in neuroglial and gliovascular interactions. Nat. Rev. Neurosci. 11, 87–99 10.1038/nrn2757 20087359

[B70] Giaume C. Orellana J. A. Abudara V. Sáez J. C. (2012). Connexin-based channels in astrocytes: how to study their properties. Methods Mol. Biol. 814, 283–303 10.1007/978-1-61779-452-0_19 22144314

[B71] Gómez-Gonzalo M. Losi G. Chiavegato A. Zonta M. Cammarota M. Brondi M. (2010). An excitatory loop with astrocytes contributes to drive neurons to seizure threshold. PLoS Biol. 8:e1000352 10.1371/journal.pbio.1000352 20405049 PMC2854117

[B72] Goubard V. Fino E. Venance L. (2011). Contribution of astrocytic glutamate and GABA uptake to corticostriatal information processing. J. Physiol. 589, 2301–2319 10.1113/jphysiol.2010.203125 21486792 PMC3098705

[B73] Grass D. Pawlowski P. G. Hirrlinger J. Papadopoulos N. Richter D. W. Kirchhoff F. (2004). Diversity of functional astroglial properties in the respiratory network. J. Neurosci. 24, 1358–1365 10.1523/JNEUROSCI.4022-03.2004 14960607 PMC6730324

[B74] Grimaldi M. Atzori M. Ray P. Alkon D. L. (2001). Mobilization of calcium from intracellular stores, potentiation of neurotransmitter-induced calcium transients, and capacitative calcium entry by 4-aminopyridine. J. Neurosci. 21, 3135–3143 11312298 10.1523/JNEUROSCI.21-09-03135.2001 PMC6762568

[B75] Haj-Yasein N. N. Jensen V. Vindedal G. F. Gundersen G. A. Klungland A. Ottersen O. P. (2011). Evidence that compromised K+ spatial buffering contributes to the epileptogenic effect of mutations in the human Kir4.1 gene (KCNJ10). Glia 59, 1635–1642 10.1002/glia.21205 21748805

[B76] Hamilton N. B. Attwell D. (2010). Do astrocytes really exocytose neurotransmitters? Nat. Rev. Neurosci. 11, 227–238 10.1038/nrn2803 20300101

[B77] Han X. Chen M. Wang F. Windrem M. Wang S. Shanz S. (2013). Forebrain engraftment by human glial progenitor cells enhances synaptic plasticity and learning in adult mice. Cell Stem Cell 12, 342–353 10.1016/j.stem.2012.12.015 23472873 PMC3700554

[B78] Hawrylak N. Chang F. L. Greenough W. T. (1993). Astrocytic and synaptic response to kindling in hippocampal subfield CA1. II. Synaptogenesis and astrocytic process increases to *in vivo* kindling. Brain Res. 603, 309–316 10.1016/0006-8993(93)91253-O 8461984

[B79] Henneberger C. Papouin T. Oliet S. H. R. Dmitri A. Rusakov D. A. (2010). Long-term potentiation depends on release of D-serine from astrocytes. Nature 463, 232–236 10.1038/nature08673 20075918 PMC2807667

[B80] Henneberger C. Rusakov D. A. (2012). Monitoring local synaptic activity with astrocytic patch pipettes. Nat. Protoc. 7, 2171–2179 10.1038/nprot.2012.140 23196973 PMC3583191

[B81] Hibino H. (2004). Differential assembly of inwardly rectifying K+ channel subunits, Kir4.1 and Kir5.1, in brain astrocytes. J. Biol. Chem. 279, 44065–44073 10.1074/jbc.M405985200 15310750

[B82] Higashi K. Fujita A. Inanobe A. Tanemoto M. Doi K. Kubo T. (2001). An inwardly rectifying K(+) channel, Kir4.1, expressed in astrocytes surrounds synapses and blood vessels in brain. Am. J. Physiol. Cell Physiol. 281, C922–C931 11502569 10.1152/ajpcell.2001.281.3.C922

[B83] Huang Y. H. Bergles D. E. (2004). Glutamate transporters bring competition to the synapse. Curr. Opin. Neurobiol. 14, 346–352 10.1016/j.conb.2004.05.007 15194115

[B84] Huang Y. H. Sinha S. R. Tanaka K. Rothstein J. D. Bergles D. E. (2004). Astrocyte glutamate transporters regulate metabotropic glutamate receptor-mediated excitation of hippocampal interneurons. J. Neurosci. 24, 4551–4559 10.1523/JNEUROSCI.5217-03.2004 15140926 PMC6729403

[B85] Innocenti B. Parpura V. Haydon P. G. (2000). Imaging extracellular waves of glutamate during calcium signaling in cultured astrocytes. J. Neurosci. 20, 1800–1808 10684881 10.1523/JNEUROSCI.20-05-01800.2000 PMC6772903

[B86] Isokawa M. McKhann G. M. (2005). Electrophysiological and morphological characterization of dentate astrocytes in the hippocampus. J. Neurobiol. 65, 125–134 10.1002/neu.20186 16114022

[B87] Jabaudon D. Scanziani M. Gahwiler B. H. Gerber U. (2000). Acute decrease in net glutamate uptake during energy deprivation. Proc. Natl. Acad. Sci. U.S.A. 97, 5610–5615 10.1073/pnas.97.10.5610 10805815 PMC25876

[B88] Jabs R. Seifert G. Steinhäuser C. (2008). Astrocytic function and its alteration in the epileptic brain. Epilepsia 49 Suppl. 2, 3–12 10.1111/j.1528-1167.2008.01488.x 18226167

[B89] Jauch R. Windmüller O. Lehmann T.-N. Heinemann U. Gabriel S. (2002). Effects of barium, furosemide, ouabaine and 4,4'-diisothiocyanatostilbene-2,2'-disulfonic acid (DIDS) on ionophoretically-induced changes in extracellular potassium concentration in hippocampal slices from rats and from patients with epilepsy. Brain Res. 925, 18–27 10.1016/S0006-8993(01)03254-1 11755897

[B90] Jones T. A. Greenough W. T. (1996). Ultrastructural evidence for increased contact between astrocytes and synapses in rats reared in a complex environment. Neurobiol. Learn. Mem. 65, 48–56 10.1006/nlme.1996.0005 8673406

[B91] Kafitz K. W. Meier S. D. Stephan J. Rose C. R. (2008). Developmental profile and properties of sulforhodamine 101–Labeled glial cells in acute brain slices of rat hippocampus. J. Neurosci. Methods 169, 84–92 10.1016/j.jneumeth.2007.11.022 18187203

[B92] Kalsi A. S. Greenwood K. Wilkin G. Butt A. M. (2004). Kir4.1 expression by astrocytes and oligodendrocytes in CNS white matter: a developmental study in the rat optic nerve. J. Anat. 204, 475–485 10.1111/j.0021-8782.2004.00288.x 15198689 PMC1571318

[B93] Kang J. Jiang L. Goldman S. A. Nedergaard M. (1998). Astrocyte-mediated potentiation of inhibitory synaptic transmission. Nat. Neurosci. 1, 683–692 10.1038/3684 10196584

[B94] Kang N. Xu J. Xu Q. Nedergaard M. Kang J. (2005). Astrocytic glutamate release-induced transient depolarization and epileptiform discharges in hippocampal CA1 pyramidal neurons. J. Neurophysiol. 94, 4121–4130 10.1152/jn.00448.2005 16162834

[B95] Karahashi Y. Goldring S. (1966). Intracellular potentials from “idle” cells in cerebral cortex of cat. Electroencephalogr. Clin. Neurophysiol. 20, 600–607 10.1016/0013-4694(66)90024-1 4161194

[B96] Karwoski C. J. Coles J. A. Lu H. K. Huang B. (1989). Current-evoked transcellular K+ flux in frog retina. J. Neurophysiol. 61, 939–952 2786057 10.1152/jn.1989.61.5.939

[B97] Kirischuk S. Kettenmann H. Verkhratsky A. (2007). Membrane currents and cytoplasmic sodium transients generated by glutamate transport in Bergmann glial cells. Pflügers Arch. Eur. J. Physiol. 454, 245–252 10.1007/s00424-007-0207-5 17273865

[B98] Kofuji P. Newman E. A. (2004). Potassium buffering in the central nervous system. Neuroscience 129, 1045–1056 10.1016/j.neuroscience.2004.06.008 15561419 PMC2322935

[B99] Kozlov A. S. Angulo M. C. Audinat E. Charpak S. (2006). Target cell-specific modulation of neuronal activity by astrocytes. Proc. Natl. Acad. Sci. U.S.A. 103, 10058–10063 10.1073/pnas.0603741103 16782808 PMC1502505

[B100] Kressin K. Kuprijanova E. Jabs R. Seifert G. Steinhäuser C. (1995). Developmental regulation of Na+ and K+ conductances in glial cells of mouse hippocampal brain slices. Glia 15, 173–187 10.1002/glia.440150210 8567069

[B101] Kroeger D. Amzica F. (2007). Hypersensitivity of the anesthesia-induced comatose brain. J. Neurosci. 27, 10597–10607 10.1523/JNEUROSCI.3440-07.2007 17898231 PMC6673173

[B102] Kucheryavykh Y. V. Kucheryavykh L. Y. Nichols C. G. Maldonado H. M. Baksi K. Reichenbach A. (2007). Downregulation of Kir4.1 inward rectifying potassium channel subunits by RNAi impairs potassium transfer and glutamate uptake by cultured cortical astrocytes. Glia 55, 274–281 10.1002/glia.20455 17091490

[B103] Kuffler S. W. Nicholls J. G. Orkand R. K. (1966). Physiological properties of glial cells in the central nervous system of amphibia. J. Neurophysiol. 29, 768–787 5966434 10.1152/jn.1966.29.4.768

[B104] Kuga N. Sasaki T. Takahara Y. Matsuki N. Ikegaya Y. (2011). Large-scale calcium waves traveling through astrocytic networks *in vivo*. J. Neurosci. 31, 2607–2614 10.1523/JNEUROSCI.5319-10.2011 21325528 PMC6623677

[B105] Langer J. Stephan J. Theis M. Rose C. R. (2012). Gap junctions mediate intercellular spread of sodium between hippocampal astrocytes *in situ*. Glia 60, 239–252 10.1002/glia.21259 22025386

[B106] Levy L. M. Warr O. Attwell D. (1998). Stoichiometry of the glial glutamate transporter GLT-1 expressed inducibly in a chinese hamster ovary cell line selected for low endogenous Na+- dependent glutamate uptake. J. Neurosci. 18, 9620–9628 9822723 10.1523/JNEUROSCI.18-23-09620.1998 PMC6793325

[B107] Lin S.-C. Bergles D. E. (2002). Physiological characteristics of NG2-expressing glial cells. J. Neurocytol. 31, 537–549 10.1023/A:1025799816285 14501222

[B108] Linden D. J. (1997). Long-term potentiation of glial synaptic currents in cerebellar culture. Neuron 18, 983–994 10.1016/S0896-6273(00)80337-2 9208865

[B109] Lüscher C. Malenka R. C. Nicoll R. A. (1998). Monitoring glutamate release during LTP with glial transporter currents. Neuron 21, 435–441 10.1016/S0896-6273(00)80552-8 9728924

[B110] Lushnikova I. Skibo G. Muller D. Nikonenko I. (2009). Synaptic potentiation induces increased glial coverage of excitatory synapses in CA1 hippocampus. Hippocampus 19, 753–762 10.1002/hipo.20551 19156853

[B111] MacFarlane S. N. Sontheimer H. (1997). Electrophysiological changes that accompany reactive gliosis *in vitro*. J. Neurosci. 17, 7316–7329 9295378 10.1523/JNEUROSCI.17-19-07316.1997 PMC6573452

[B112] MacVicar B. A. Feighan D. Brown A. Ransom B. (2002). Intrinsic optical signals in the rat optic nerve: role for K(+) uptake via NKCC1 and swelling of astrocytes. Glia 37, 114–123 10.1002/glia.10023 11754210

[B113] MacVicar B. A. Hochman D. (1991). Imaging of synaptically evoked intrinsic optical signals in hippocampal slices. J. Neurosci. 11, 1458–1469 1851222 10.1523/JNEUROSCI.11-05-01458.1991 PMC6575307

[B114] Maldonado P. P. Vélez-Fort M. Levavasseur F. Angulo M. C. (2013). Oligodendrocyte precursor cells are accurate sensors of local K+ in mature gray matter. J. Neurosci. 33, 2432–2442 10.1523/JNEUROSCI.1961-12.2013 23392672 PMC6619152

[B115] Matthias K. Kirchhoff F. Seifert G. Hüttmann K. Matyash M. Kettenmann H. (2003). Segregated expression of AMPA-type glutamate receptors and glutamate transporters defines distinct astrocyte populations in the mouse hippocampus. J. Neurosci. 23, 1750–1758 12629179 10.1523/JNEUROSCI.23-05-01750.2003 PMC6741945

[B116] Meeks J. P. Mennerick S. (2007). Astrocyte membrane responses and potassium accumulation during neuronal activity. Hippocampus 17, 1100–1108 10.1002/hipo.20344 17853441

[B117] Même W. Vandecasteele M. Giaume C. Venance L. (2009). Electrical coupling between hippocampal astrocytes in rat brain slices. Neurosci. Res. 63, 236–243 10.1016/j.neures.2008.12.008 19167439

[B118] Mennerick S. Zorumski C. F. (1994). Glial contributions to excitatory neurotransmission in cultured hippocampal cells. Nature 368, 59–62 10.1038/368059a0 7906399

[B119] Minelli A. Barbaresi P. Reimer R. Edwards R. Conti F. (2001). The glial glutamate transporter GLT-1 is localized both in the vicinity of and at distance from axon terminals in the rat cerebral cortex. Neuroscience 108, 51–59 10.1016/S0306-4522(01)00375-X 11738130

[B120] Mishima T. Hirase H. (2010). *In vivo* intracellular recording suggests that gray matter astrocytes in mature cerebral cortex and hippocampus are electrophysiologically homogeneous. J. Neurosci. 30, 3093–3100 10.1523/JNEUROSCI.5065-09.2010 20181606 PMC6633951

[B121] Mishima T. Sakatani S. Hirase H. (2007). Intracellular labeling of single cortical astrocytes *in vivo*. J. Neurosci. Methods 166, 32–40 10.1016/j.jneumeth.2007.06.021 17686526

[B122] Molenaar R. J. (2011). Ion channels in glioblastoma. Isrn Neurol. 2011:590249 10.5402/2011/590249 22389824 PMC3263536

[B123] Müller M. Somjen G. G. (2000). Na+ and K+ concentrations, extra- and intracellular voltages, and the effect of TTX in hypoxic rat hippocampal slices. J. Neurophysiol. 83, 735–745 10669489 10.1152/jn.2000.83.2.735

[B124] Murphy T. H. Wier W. G. (1993). Rapid communication between neurons and astrocytes in primary cortical cultures. J. Neurosci. 13, 2672–2679 8501531 10.1523/JNEUROSCI.13-06-02672.1993 PMC6576490

[B125] Navarrete M. Araque A. (2008). Endocannabinoids mediate neuron-astrocyte communication. Neuron 57, 883–893 10.1016/j.neuron.2008.01.029 18367089

[B126] Navarrete M. Araque A. (2010). Endocannabinoids potentiate synaptic transmission through stimulation of astrocytes. Neuron 68, 113–126 10.1016/j.neuron.2010.08.043 20920795

[B127] Navarrete M. Perea G. Maglio L. Pastor J. García de Sola R. Araque A. (2012). Astrocyte calcium signal and gliotransmission in human brain tissue. Cereb. Cortex 23, 1240–1246 10.1093/cercor/bhs122 22581850

[B128] Nedergaard M. Verkhratsky A. (2012). Artifact versus reality–how astrocytes contribute to synaptic events. Glia 60, 1013–1023 10.1002/glia.22288 22228580 PMC3340515

[B129] Neusch C. Papadopoulos N. Müller M. Maletzki I. Winter S. M. Hirrlinger J. (2006). Lack of the Kir4.1 channel subunit abolishes K+ buffering properties of astrocytes in the ventral respiratory group: impact on extracellular K+ regulation. J. Neurophysiol. 95, 1843–1852 10.1152/jn.00996.2005 16306174

[B130] Newman E. Reichenbach A. (1996). The Müller cell: a functional element of the retina. Trends Neurosci. 19, 307–312 10.1016/0166-2236(96)10040-0 8843598

[B131] Newman E. A. (2003). New roles for astrocytes: regulation of synaptic transmission. Trends Neurosci. 26, 536–542 10.1016/S0166-2236(03)00237-6 14522146

[B132] Nixdorf-Bergweiler B. E. Albrecht D. Heinemann U. (1994). Developmental changes in the number, size, and orientation of GFAP-positive cells in the CA1 region of rat hippocampus. Glia 12, 180–195 10.1002/glia.440120304 7851987

[B133] O'Connor E. R. Sontheimer H. Spencer D. D. de Lanerolle N. C. (1998). Astrocytes from human hippocampal epileptogenic foci exhibit action potential-like responses. Epilepsia 39, 347–354 10.1111/j.1528-1157.1998.tb01386.x 9578024

[B134] Oliet S. H. Piet R. Poulain D. A. (2001). Control of glutamate clearance and synaptic efficacy by glial coverage of neurons. Science 292, 923–926 10.1126/science.1059162 11340204

[B135] Oliet S. H. R. Bonfardin V. D. J. (2010). Morphological plasticity of the rat supraoptic nucleus–cellular consequences. Eur. J. Neurosci. 32, 1989–1994 10.1111/j.1460-9568.2010.07514.x 21143653

[B136] Olsen M. (2012). Examining potassium channel function in astrocytes, in Methods in Molecular Biology, ed Walker J. M. (Clifton, NJ: Humana Press), 265 10.1007/978-1-61779-452-0_18 22144313

[B137] Olsen M. L. Higashimori H. Campbell S. L. Hablitz J. J. Sontheimer H. (2006). Functional expression of Kir4.1 channels in spinal cord astrocytes. Glia 53, 516–528 10.1002/glia.20312 16369934 PMC2553202

[B138] Olsen M. L. Sontheimer H. (2008). Functional implications for Kir4.1 channels in glial biology: from K+ buffering to cell differentiation. J. Neurochem. 107, 589–601 10.1111/j.1471-4159.2008.05615.x 18691387 PMC2581639

[B139] Orkand R. K. Nicholls J. G. Kuffler S. W. (1966). Effect of nerve impulses on the membrane potential of glial cells in the central nervous system of amphibia. J. Neurophysiol. 29, 788–806 5966435 10.1152/jn.1966.29.4.788

[B140] Owe S. G. Marcaggi P. Attwell D. (2006). The ionic stoichiometry of the GLAST glutamate transporter in salamander retinal glia. J. Physiol. 577, 591–599 10.1113/jphysiol.2006.116830 17008380 PMC1890427

[B141] Panatier A. Vallée J. Haber M. Murai K. K. Lacaille J.-C. Robitaille R. (2011). Astrocytes are endogenous regulators of basal transmission at central synapses. Cell 146, 785–798 10.1016/j.cell.2011.07.022 21855979

[B142] Pannasch U. Derangeon M. Chever O. Rouach N. (2012a). Astroglial gap junctions shape neuronal network activity. Commun. Integr. Biol. 5, 248–254 10.4161/cib.19410 22896785 PMC3419107

[B143] Pannasch U. Sibille J. Rouach N. (2012b). Dual electrophysiological recordings of synaptically-evoked astroglial and neuronal responses in acute hippocampal slices. J. Vis. Exp. 69:e4418 10.3791/4418 23222635 PMC3564483

[B144] Pannasch U. Vargová L. Reingruber J. Ezan P. Holcman D. Giaume C. (2011). Astroglial networks scale synaptic activity and plasticity. Proc. Natl. Acad. Sci. U.S.A. 108, 8467–8472 10.1073/pnas.1016650108 21536893 PMC3100942

[B145] Parpura V. Haydon P. G. (2000). Physiological astrocytic calcium levels stimulate glutamate release to modulate adjacent neurons. Proc. Natl. Acad. Sci. U.S.A. 97, 8629–8634 10.1073/pnas.97.15.8629 10900020 PMC26999

[B146] Parri H. R. Gould T. M. Crunelli V. (2010). Sensory and cortical activation of distinct glial cell subtypes in the somatosensory thalamus of young rats. Eur. J. Neurosci. 32, 29–40 10.1111/j.1460-9568.2010.07281.x 20608967 PMC2909395

[B147] Päsler D. Gabriel S. Heinemann U. (2007). Two-pore-domain potassium channels contribute to neuronal potassium release and glial potassium buffering in the rat hippocampus. Brain Res. 1173, 14–26 10.1016/j.brainres.2007.07.013 17850772

[B148] Pellerin L. Magistretti P. J. (1994). Glutamate uptake into astrocytes stimulates aerobic glycolysis: a mechanism coupling neuronal activity to glucose utilization. Proc. Natl. Acad. Sci. U.S.A. 91, 10625–10629 10.1073/pnas.91.22.10625 7938003 PMC45074

[B149] Pellerin L. Magistretti P. J. (2012). Sweet sixteen for ANLS. J. Cereb. Blood Flow Metab. Off. J. Int. Soc. Cereb. Blood Flow Metab. 32, 1152–1166 10.1038/jcbfm.2011.149 22027938 PMC3390819

[B150] Perea G. Navarrete M. Araque A. (2009). Tripartite synapses: astrocytes process and control synaptic information. Trends Neurosci. 32, 421–431 10.1016/j.tins.2009.05.001 19615761

[B151] Petravicz J. Fiacco T. A. McCarthy K. D. (2008). Loss of IP3 receptor-dependent Ca2+ increases in hippocampal astrocytes does not affect baseline CA1 pyramidal neuron synaptic activity. J. Neurosci. 28, 4967–4973 10.1523/JNEUROSCI.5572-07.2008 18463250 PMC2709811

[B152] Phillips C. G. (1956). Intracellular records from Betz cells in the cat. Q. J. Exp. Physiol. Cogn. Med. Sci. 41, 58–69 13485327 10.1113/expphysiol.1956.sp001163

[B153] Piet R. Vargová L. Syková E. Poulain D. A. Oliet S. H. R. (2004). Physiological contribution of the astrocytic environment of neurons to intersynaptic crosstalk. Proc. Natl. Acad. Sci. U.S.A. 101, 2151–2155 10.1073/pnas.0308408100 14766975 PMC357067

[B154] Poolos N. P. Mauk M. D. Kocsis J. D. (1987). Activity-evoked increases in extracellular potassium modulate presynaptic excitability in the CA1 region of the hippocampus. J. Neurophysiol. 58, 404–416 3655875 10.1152/jn.1987.58.2.404

[B155] Proper E. A Hoogland G. Kappen S. M. Jansen G. H. Rensen M. G. A Schrama L. H. (2002). Distribution of glutamate transporters in the hippocampus of patients with pharmaco-resistant temporal lobe epilepsy. Brain J. Neurol. 125, 32–43 10.1093/brain/awf001 11834591

[B156] Pumain R. Heinemann U. (1985). Stimulus- and amino acid-induced calcium and potassium changes in rat neocortex. J. Neurophysiol. 53, 1–16 2857775 10.1152/jn.1985.53.1.1

[B157] Richardson W. D. Young K. M. Tripathi R. B. McKenzie I. (2011). NG2-glia as multipotent neural stem cells: fact or fantasy? Neuron 70, 661–673 10.1016/j.neuron.2011.05.013 21609823 PMC3119948

[B158] Ransom B. R. Goldring S. (1973). Slow hyperpolarization in cells presumed to be glia in cerebral cortex of cat. J. Neurophysiol. 36, 879–892 4805017 10.1152/jn.1973.36.5.879

[B159] Ransom B. R. Yamate C. L. Connors B. W. (1985). Activity-dependent shrinkage of extracellular space in rat optic nerve: a developmental study. J. Neurosci. 5, 532–535 3973681 10.1523/JNEUROSCI.05-02-00532.1985 PMC6565184

[B160] Ransom C. B. O'Neal J. T. Sontheimer H. (2001). Volume-activated chloride currents contribute to the resting conductance and invasive migration of human glioma cells. J. Neurosci. 21, 7674–7683 11567057 10.1523/JNEUROSCI.21-19-07674.2001 PMC6762888

[B161] Ransom C. B. Ransom B. R. Sontheimer H. (2000). Activity-dependent extracellular K+ accumulation in rat optic nerve: the role of glial and axonal Na+ pumps. J. Physiol. 522(Pt 3), 427–442 10.1111/j.1469-7793.2000.00427.x 10713967 PMC2269766

[B162] Raz-prag D. Grimes W. N. Fariss R. N. Vijayasarathy C. Campos M. M. (2010). Probing potassium channel function *in vivo* by intracellular delivery of antibodies in a rat model of retinal neurodegeneration. Proc. Natl. Acad. Sci. U.S.A. 107, 12710 10.1073/pnas.0913472107 20616020 PMC2906585

[B163] Rothstein J. D. Martin L. Levey A. I. Dykes-Hoberg M. Jin L. Wu D. (1994). Localization of neuronal and glial glutamate transporters. Neuron 13, 713–725 10.1016/0896-6273(94)90038-8 7917301

[B164] Rouach N. Koulakoff A. Abudara V. Willecke K. Giaume C. (2008). Astroglial metabolic networks sustain hippocampal synaptic transmission. Science 322, 1551–1555 10.1126/science.1164022 19056987

[B165] Roux L. Benchenane K. Rothstein J. D. Bonvento G. Giaume C. (2011). Plasticity of astroglial networks in olfactory glomeruli. Proc. Natl. Acad. Sci. U.S.A. 108, 18442–18446 10.1073/pnas.1107386108 21997206 PMC3214998

[B166] Ruminot I. Gutiérrez R. Peña-Münzenmayer G. Añazco C. Sotelo-Hitschfeld T. Lerchundi R. (2011). NBCe1 mediates the acute stimulation of astrocytic glycolysis by extracellular K+. J. Neurosci. 31, 14264–14271 10.1523/JNEUROSCI.2310-11.2011 21976511 PMC3200293

[B168] Santello M. Bezzi P. Volterra A. (2011). TNFα controls glutamatergic gliotransmission in the hippocampal dentate gyrus. Neuron 69, 988–1001 10.1016/j.neuron.2011.02.003 21382557

[B169] Santello M. Volterra A. (2012). TNFα in synaptic function: switching gears. Trends Neurosci. 35, 638–647 10.1016/j.tins.2012.06.001 22749718

[B170] Schools G. P. Zhou M. Kimelberg H. K. (2006). Development of gap junctions in hippocampal astrocytes: evidence that whole cell electrophysiological phenotype is an intrinsic property of the individual cell. J. Neurophysiol. 96, 1383–1392 10.1152/jn.00449.2006 16775204

[B171] Schröder W. Hinterkeuser S. Seifert G. Schramm J. Jabs R. Wilkin G. P. (2000). Functional and molecular properties of human astrocytes in acute hippocampal slices obtained from patients with temporal lobe epilepsy. Epilepsia 41 Suppl. 6, S181–S184 10.1111/j.1528-1157.2000.tb01578.x 10999541

[B172] Schröder W. Seifert G. Hüttmann K. Hinterkeuser S. Steinhäuser C. (2002). AMPA receptor-mediated modulation of inward rectifier K+ channels in astrocytes of mouse hippocampus. Mol. Cell. Neurosci. 19, 447–458 10.1006/mcne.2001.1080 11906215

[B173] Scimemi A. Tian H. Diamond J. S. (2009). Neuronal transporters regulate glutamate clearance, NMDA receptor activation, and synaptic plasticity in the hippocampus. J. Neurosci. 29, 14581–14595 10.1523/JNEUROSCI.4845-09.2009 19923291 PMC2853250

[B174] Seifert G. Hüttmann K. Binder D. K. Hartmann C. Wyczynski A. Neusch C. (2009). Analysis of astroglial K+ channel expression in the developing hippocampus reveals a predominant role of the Kir4.1 subunit. J. Neurosci. 29, 7474–7488 10.1523/JNEUROSCI.3790-08.2009 19515915 PMC6665420

[B175] Seigneur J. Kroeger D. Nita D. A. Amzica F. (2006). Cholinergic action on cortical glial cells *in vivo*. Cereb. Cortex 16, 655–668 10.1093/cercor/bhj011 16093563

[B176] Shigetomi E. Bowser D. N. Sofroniew M. V. Khakh B. S. (2008). Two forms of astrocyte calcium excitability have distinct effects on NMDA receptor-mediated slow inward currents in pyramidal neurons. J. Neurosci. 28, 6659–6663 10.1523/JNEUROSCI.1717-08.2008 18579739 PMC2866443

[B177] Solessio E. Linn D. M. Perlman I. Lasater E. M. (2000). Characterization with barium of potassium currents in turtle retinal Müller cells. J. Neurophysiol. 83, 418–430 10634884 10.1152/jn.2000.83.1.418

[B178] Somjen G. G. (1975). Electrophysiology of neuroglia. Annu. Rev. Physiol. 37, 163–190 10.1146/annurev.ph.37.030175.001115 1092250

[B179] Somjen G. G. (2002). Ion regulation in the brain: implications for pathophysiology. Neuroscientist 8, 254–267 10.1177/10758402008003011 12061505

[B180] Somjen G. G. Kager H. Wadman W. J. (2008). Computer simulations of neuron-glia interactions mediated by ion flux. J. Comput. Neurosci. 25, 349–365 10.1007/s10827-008-0083-9 18297383

[B181] Sontheimer H. (1994). Voltage-dependent ion channels in glial cells. Glia 11, 156–172 10.1002/glia.440110210 7523291

[B182] Sontheimer H. Black J. A. Waxman S. G. (1996). Voltage-gated Na+ channels in glia: properties and possible functions. Trends Neurosci. 19, 325–331 10.1016/0166-2236(96)10039-4 8843601

[B183] Sontheimer H. Waxman S. G. (1992). Ion channels in spinal cord astrocytes *in vitro*. II. Biophysical and pharmacological analysis of two Na+ current types. J. Neurophysiol. 68, 1001–1011 1331355 10.1152/jn.1992.68.4.1001

[B184] Sontheimer H. Waxman S. G. (1993). Expression of voltage-activated ion channels by astrocytes and oligodendrocytes in the hippocampal slice. J. Neurophysiol. 70, 1863–1873 7507520 10.1152/jn.1993.70.5.1863

[B185] Steinhäuser C. Jabs R. Kettenmann H. (1994). Properties of GABA and glutamate responses in identified glial cells of the mouse hippocampal slice. Hippocampus 4, 19–35 10.1002/hipo.450040105 7914797

[B186] Steinhäuser C. Seifert G. Deitmer J. W. (2013). Physiology of astrocytes: ions channels and ion transporters, in Neuroglia, eds Kettenmann H. Ransom B. R (New York, NY: Oxford University Press), 185

[B187] Sugaya E. Goldring S. O'Leary J. L. (1964). Intracellular potentials associated with direct cortical response and seizure discharge in cat. Electroencephalogr. Clin. Neurophysiol. 17, 661–669 10.1016/0013-4694(64)90234-2 14240859

[B188] Sun W. McConnell E. Pare J.-F. Xu Q. Chen M. Peng W. (2013). Glutamate-dependent neuroglial calcium signaling differs between young and adult brain. Science 339, 197–200 10.1126/science.1226740 23307741 PMC3569008

[B189] Tzingounis A. V. Wadiche J. I. (2007). Glutamate transporters: confining runaway excitation by shaping synaptic transmission. Nat. Rev. Neurosci. 8, 935–947 10.1038/nrn2274 17987031

[B190] Unichenko P. Myakhar O. Kirischuk S. (2012). Intracellular Na+ concentration influences short-term plasticity of glutamate transporter-mediated currents in neocortical astrocytes. Glia 60, 605–614 10.1002/glia.22294 22279011

[B191] Verkhratsky A. Kettenmann H. (1996). Calcium signalling in glial cells. Trends Neurosci. 19, 346–352 10.1016/0166-2236(96)10048-5 8843604

[B192] Verkhratsky A. Steinhäuser C. (2000). Ion channels in glial cells. Brain Res. Brain Res. Rev. 32, 380–412 10.1016/S0165-0173(99)00093-4 10760549

[B193] Wallraff A. Köhling R. Heinemann U. Theis M. Willecke K. Steinhäuser C. (2006). The Impact of Astrocytic Gap Junctional Coupling on Potassium Buffering in the Hippocampus. J. Neurosci. 26, 5438–5447 10.1523/JNEUROSCI.0037-06.2006 16707796 PMC6675300

[B194] Wallraff A. Odermatt B. Willecke K. Steinhäuser C. (2004). Distinct types of astroglial cells in the hippocampus differ in gap junction coupling. Glia 48, 36–43 10.1002/glia.20040 15326613

[B195] Wang X. Lou N. Xu Q. Tian G.-F. Peng W. G. Han X. (2006). Astrocytic Ca2+ signaling evoked by sensory stimulation *in vivo*. Nat. Neurosci. 9, 816–823 10.1038/nn1703 16699507

[B196] Wenzel J. Lammert G. Meyer U. Krug M. (1991). The influence of long-term potentiation on the spatial relationship between astrocyte processes and potentiated synapses in the dentate gyrus neuropil of rat brain. Brain Res. 560, 122–131 10.1016/0006-8993(91)91222-M 1760721

[B197] Woo D. H. Han K.-S. Shim J. W. Yoon B.-E. Kim E. Bae J. Y. (2012). TREK-1 and Best1 channels mediate fast and slow glutamate release in astrocytes upon GPCR activation. Cell 151, 25–40 10.1016/j.cell.2012.09.005 23021213

[B198] Xiong Z. Q. Stringer J. L. (2000). Sodium pump activity, not glial spatial buffering, clears potassium after epileptiform activity induced in the dentate gyrus. J. Neurophysiol. 83, 1443–1451 10712471 10.1152/jn.2000.83.3.1443

[B199] Xu G. Wang W. Kimelberg H. K. Zhou M. (2010). Electrical coupling of astrocytes in rat hippocampal slices under physiological and simulated ischemic conditions. Glia 58, 481–493 10.1002/glia.20939 19795502

[B200] Zhang H. Xin W. Dougherty P. M. (2009). Synaptically evoked glutamate transporter currents in spinal dorsal horn astrocytes. Mol. Pain 5, 36 10.1186/1744-8069-5-36 19570219 PMC2713213

[B201] Zhou M. (2005). Development of GLAST(+) astrocytes and NG2(+) glia in rat hippocampus CA1: mature astrocytes are electrophysiologically passive. J. Neurophysiol. 95, 134–143 10.1152/jn.00570.2005 16093329

[B202] Zhou M. Xu G. Xie M. Zhang X. Schools G. P. Ma L. (2009). TWIK-1 and TREK-1 are potassium channels contributing significantly to astrocyte passive conductance in rat hippocampal slices. J. Neurosci. 29, 8551–8564 10.1523/JNEUROSCI.5784-08.2009 19571146 PMC6665656

